# Activation of Ran GTPase by a *Legionella* Effector Promotes Microtubule Polymerization, Pathogen Vacuole Motility and Infection

**DOI:** 10.1371/journal.ppat.1003598

**Published:** 2013-09-19

**Authors:** Eva Rothmeier, Gudrun Pfaffinger, Christine Hoffmann, Christopher F. Harrison, Heinrich Grabmayr, Urska Repnik, Mandy Hannemann, Stefan Wölke, Andreas Bausch, Gareth Griffiths, Annette Müller-Taubenberger, Aymelt Itzen, Hubert Hilbi

**Affiliations:** 1 Max von Pettenkofer-Institute, Department of Medicine, Ludwig-Maximilians Universität München, München, Germany; 2 Institute of Molecular and Cellular Biophysics, Department of Physics, Technische Universität München, Garching, Germany; 3 Department of Molecular Biosciences, University of Oslo, Oslo, Norway; 4 Center for Integrated Protein Science Munich, Department of Chemistry, Technische Universität München, Garching, Germany; 5 Institute for Anatomy and Cell Biology, Department of Medicine, Ludwig-Maximilians Universität München, München, Germany; Osaka University, Japan

## Abstract

The causative agent of Legionnaires' disease, *Legionella pneumophila*, uses the Icm/Dot type IV secretion system (T4SS) to form in phagocytes a distinct “*Legionella*-containing vacuole” (LCV), which intercepts endosomal and secretory vesicle trafficking. Proteomics revealed the presence of the small GTPase Ran and its effector RanBP1 on purified LCVs. Here we validate that Ran and RanBP1 localize to LCVs and promote intracellular growth of *L. pneumophila*. Moreover, the *L. pneumophila* protein LegG1, which contains putative RCC1 Ran guanine nucleotide exchange factor (GEF) domains, accumulates on LCVs in an Icm/Dot-dependent manner. *L. pneumophila* wild-type bacteria, but not strains lacking LegG1 or a functional Icm/Dot T4SS, activate Ran on LCVs, while purified LegG1 produces active Ran(GTP) in cell lysates. *L. pneumophila* lacking *legG1* is compromised for intracellular growth in macrophages and amoebae, yet is as cytotoxic as the wild-type strain. A downstream effect of LegG1 is to stabilize microtubules, as revealed by conventional and stimulated emission depletion (STED) fluorescence microscopy, subcellular fractionation and Western blot, or by microbial microinjection through the T3SS of a *Yersinia* strain lacking endogenous effectors. Real-time fluorescence imaging indicates that LCVs harboring wild-type *L. pneumophila* rapidly move along microtubules, while LCVs harboring Δ*legG1* mutant bacteria are stalled. Together, our results demonstrate that Ran activation and RanBP1 promote LCV formation, and the Icm/Dot substrate LegG1 functions as a bacterial Ran activator, which localizes to LCVs and promotes microtubule stabilization, LCV motility as well as intracellular replication of *L. pneumophila*.

## Author Summary

*Legionella pneumophila* is an environmental bacterium that grows within free-living amoebae and, upon inhalation, in human lung macrophages, thus causing the severe pneumonia Legionnaires' disease. Within amoebae or macrophages the bacteria form a distinct membrane-bound replication niche, the “*Legionella*-containing vacuole” (LCV). To this end, *L. pneumophila* injects via a dedicated secretion apparatus about 300 different “effector” proteins directly into host cells, where they interfere with cellular processes. LCV formation is poorly understood, and the function and targets of most bacterial effector proteins are unknown. In this study, we characterize an *L. pneumophila* effector protein that activates the small host GTPase Ran, which is essential for crucial cellular processes, such as spindle assembly and cytokinesis, nucleo-cytoplasmic transport, as well as nuclear envelope formation. We discovered that Ran promotes intracellular replication of *L. pneumophila* and its activation on the LCV membrane by LegG1 causes the polymerization of microtubules, along which cellular vesicles as well as LCVs move within cells. Our study defines a novel strategy how pathogenic bacteria subvert host processes to promote intracellular survival and replication.

## Introduction

The amoebae-resistant environmental bacterium *Legionella pneumophila* is the causative agent of a severe pneumonia termed Legionnaires' disease [1], [2]. In free-living amoebae as well as in macrophages of the innate immune system, *L. pneumophila* employs an apparently conserved mechanism to form a replication-permissive membrane-bound compartment, the “*Legionella*-containing vacuole” (LCV) [3], [4], [5]. LCVs avoid fusion with bactericidal lysosomes, and instead interact in a bi-phasic process with early secretory vesicles budding from endoplasmic reticulum (ER) exit sites and with the ER. Microtubules play a role in the initial trafficking events of LCVs, prior to the acquisition of the early secretory vesicle marker GFP-HDEL and the resident ER marker calnexin-GFP [6]. A proteomics analysis of purified intact LCVs revealed more than 560 host proteins, including α- and β-tubulin, as well as a number of small GTPases and GTPase-interacting factors [7]. In addition to Arf1 and Rab GTPases implicated in the secretory and endosomal vesicle trafficking pathways, Ran and its effector Ran binding protein 1 (RanBP1) were identified in this study as LCV host components.

The small GTPase Ran is implicated in a variety of cellular processes, such as nuclear pore translocation [8], or mitotic spindle assembly and post-mitotic nuclear envelope formation [9], [10]. Furthermore, Ran plays an important role in cytoplasmic events involving non-centrosomal microtubules, e.g. endocytic receptor trafficking and retrograde signaling along microtubules in nerve axons [11]. Ran can be activated by a nuclear (or in mitotic cells: chromatin-bound) Ran guanine nucleotide exchange factor (GEF) termed regulator of chromosome condensation 1 (RCC1) [12]. Ran(GTP) is inactivated by the cytoplasmic Ran GTPase-activating protein 1 (RanGAP1) in concert with RanBP1 harboring a Ran(GTP)-binding domain [11].

The common mechanism of Ran activity involves sequestration of a transport complex compound by Ran(GTP), which is liberated upon GTP hydrolysis. Thus the displacement of Ran leads to the assembly of functional transport complexes and process activation [13]. A prominent example of this mechanism is the direct binding of Ran(GTP) to β-importin, preventing the formation (or leading to disassembly) of cargo transport complexes during nucleo-cytoplasmic transport, axonal retrograde signaling and post-mitotic nuclear membrane reconstitution. In addition, Ran has been implicated in endocytic receptor trafficking [14] and cytoplasmic organization of non-centrosomal microtubules [15]. A role for Ran in pathogen vacuole formation has not yet been described.

The formation of the *Legionella*-containing pathogen vacuole requires the Icm/Dot type IV secretion system (T4SS), which translocates at least 275 different “effector proteins” into eukaryotic cells [4], [16], [17]. The function of most Icm/Dot substrates is unknown, yet recent studies by several groups shed light on the intricate manner by which some effector proteins modulate small host GTPases. To this end, *L. pneumophila* produces an Arf1 GEF termed RalF [18] and devotes as many as six different translocated effectors to subvert the function of Rab1 [5]. SidM (*alias* DrrA) functions as a Rab1 GEF and guanine dissociation inhibitor (GDI) displacement factor (GDF) [19], [20], [21], [22], while LepB deactivates Rab1 through its Rab1 GAP activity [23]. Interestingly, SidM also acts as an adenylyl transferase by covalently attaching AMP to Rab1 [24], [25], and AnkX attaches a phosphocholine moiety to Rab1 [25], [26]. The covalent adenylylation or phosphocholination modifications are reversible, and the corresponding deadenylylation or dephosphocholination reactions are catalyzed by the effector proteins SidD [27], [28] or Lem3 [29], [30], respectively. Finally, the Icm/Dot substrate LidA supports the GEF activity of SidM [20] and binds with immense affinity to activated Rab1 [31]. SidM, but not SidD or RalF, anchors to the LCV membrane by binding with high affinity to the phosphoinositide (PI) lipid phosphatidylinositol-4-phosphate (PtdIns(4)*P*) [32], [33], [34].

Bacterial proteins targeting the small GTPase Ran have not been characterized, yet the Icm/Dot-translocated *L. pneumophila* protein LegG1 (*Legionella* eukaryotic gene G1; lpg1976) shows amino acid sequence homology to the Ran GEF RCC1 [35], [36], [37]. LegG1 (*alias* PieG) is encoded in the Pie (Plasticity island of effectors) gene cluster and localizes to small vesicle-like structures in eukaryotic cells upon ectopic production [37]. LegG1/PieG contains a C-terminal CAAX tetrapeptide motif, which is lipidated by the host prenylation machinery to facilitate targeting of the bacterial protein to host membranes [38]. Mutation of the conserved cysteine to serine, as well as treatment with the isoprenoid biosynthesis inhibitor mevastatin or with a geranylgeranyltransferase inhibitor abolished membrane localization of ectopically produced LegG1, suggesting that prenylation is the major if not sole membrane-targeting determinant [38]. The function of LegG1 in *L. pneumophila*-infected host cells is so far unknown. Here, we demonstrate that LegG1 acts as a Ran activator in infected cells and cell lysates. Moreover, the activation of Ran on LCVs promotes microtubule stabilization, LCV motility and intracellular replication of *L. pneumophila*.

## Results

### The small GTPase Ran and the Icm/Dot substrate LegG1 localize to LCVs

**Figure 1:**
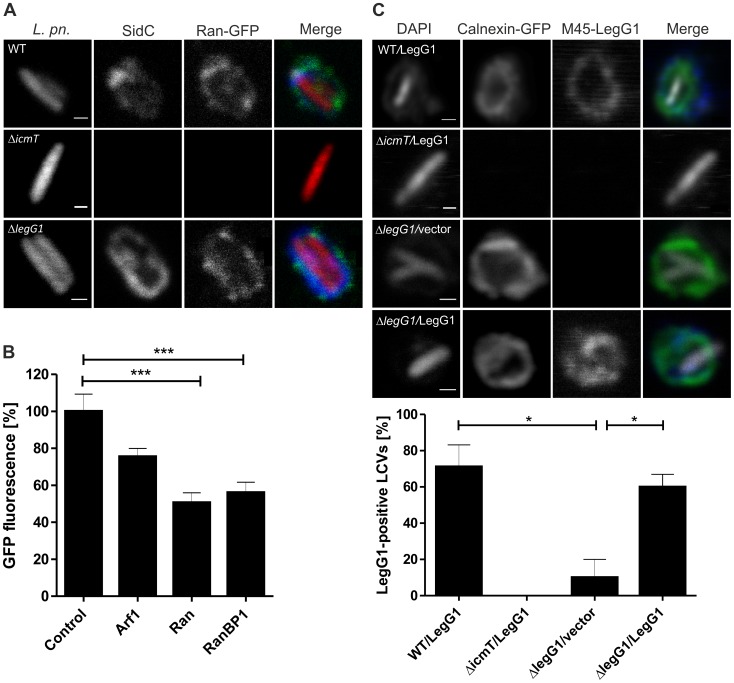
The small GTPase Ran and the Icm/Dot substrate LegG1 localize to LCVs. (**A**) Ran accumulates on LCVs. *D. discoideum* producing RanA-GFP was infected (MOI 50, 1 h) with DsRed-producing *L. pneumophila* wild-type, Δ*legG1* or Δ*icmT* harboring pSW001 and immuno-stained for the LCV membrane marker SidC. LCVs in lysates of infected cells are shown. (**B**) Depletion of Ran or RanBP1 inhibits intracellular growth of *L. pneumophila*. A549 lung epithelial carcinoma cells were treated with AllStars siRNA (negative control) or with siRNA oligonucleotides targeting Ran, RanBP1 or Arf1 (positive control) for 2 d, and intracellular replication of GFP-producing *L. pneumophila* harboring pNT28 was quantified by fluorescence measurements after 24 h. Data represent mean and standard deviation of three independent experiments considering the 3 most effective out of 4 different oligonucleotides (Fig. S1B). Student's t-test; ***, *p*<0.001. (**C**) Icm/Dot-dependent localization of M45-LegG1 on LCVs in cell homogenates. *D. discoideum* producing calnexin-GFP was infected (MOI 50) with *L. pneumophila* wild-type, Δ*icmT* or Δ*legG1* harboring pSU19 (M45-LegG1) or with Δ*legG1* harboring pCR033 (vector), homogenized and immuno-stained with an anti-M45 antibody and with DAPI. The percentage of M45-LegG1-positive LCVs (n = 100/strain, 3 independent experiments) was scored in lysates of infected cells (*, *p*<0.05). Bars (A, C), 0.5 µm.

The small GTPase Ran and its effector RanBP1 have been identified on purified LCVs by proteomic analysis [7], [39]. To directly investigate the presence of these proteins on LCVs by fluorescence microscopy, *Dictyostelium discoideum* producing the corresponding GFP fusion proteins was infected with red fluorescent *L. pneumophila*. Ran was found to localize to the LCV membrane in *D. discoideum* infected with *L. pneumophila* wild-type or Δ*legG1* but not with Δ*icmT* mutant bacteria (Figure 1A). Moreover, RanBP1 localized to LCVs harboring wild-type *L. pneumophila* (see below). These results confirm the proteomic data and show that Ran and RanBP1 localize to LCVs in an Icm/Dot-dependent manner.

To assess whether Ran or RanBP1 play a role for intracellular replication of *L. pneumophila* we depleted the proteins by RNA interference. To this end, A549 lung epithelial cells were treated with siRNA oligonucleotides targeting Ran, RanBP1 or, as a positive control, Arf1, and intracellular replication of *L. pneumophila* was monitored over 24 h (Figure 1B). Upon depletion of either Ran or RanBP1 the number of intracellular *L. pneumophila* was reduced two-fold, indicating that the small GTPase as well as its effector RanBP1 are required for efficient intracellular growth of *L. pneumophila*. As expected, the depletion of Arf1 also reduced intracellular growth of *L. pneumophila*, albeit less efficiently than depletion of Ran or RanBP1. The treatment with siRNA oligonucleotides efficiently depleted Ran or RanBP1, yet had no effect on A549 cell viability (Figure S1). Therefore, the depletion of Ran or RanBP1 impedes the intracellular replication of *L. pneumophila* without dramatically affecting the host cell physiology.

The *legG1* gene is conserved among the *L. pneumophila* strains sequenced to date (Philadelphia-1, Paris, Lens, Corby, Alcoy, 130b/AA100, Lorraine, HL06041035), but apparently not present in other *Legionella* spp. LegG1 is translocated by the Icm/Dot T4SS as a TEM-β-lactamase fusion protein into J774 or RAW264.7 macrophages ([36]; Figure S2), or as an adenylate cyclase fusion protein into CHO cells [37]. Upon infection of *D. discoideum* producing the ER/LCV marker calnexin-GFP with red fluorescent *L. pneumophila* producing M45-tagged LegG1, the effector protein accumulated in an Icm/Dot-dependent manner on the LCV membrane (Figure 1C). Under these conditions, approximately 60–70% of the LCVs scored positive for M45-LegG1. In summary, Ran, RanBP1 and LegG1 all accumulate on the LCV membrane in an Icm/Dot-dependent manner.

### *L. pneumophila* wild-type but not Δ*legG1* activates Ran on LCVs

**Figure 2:**
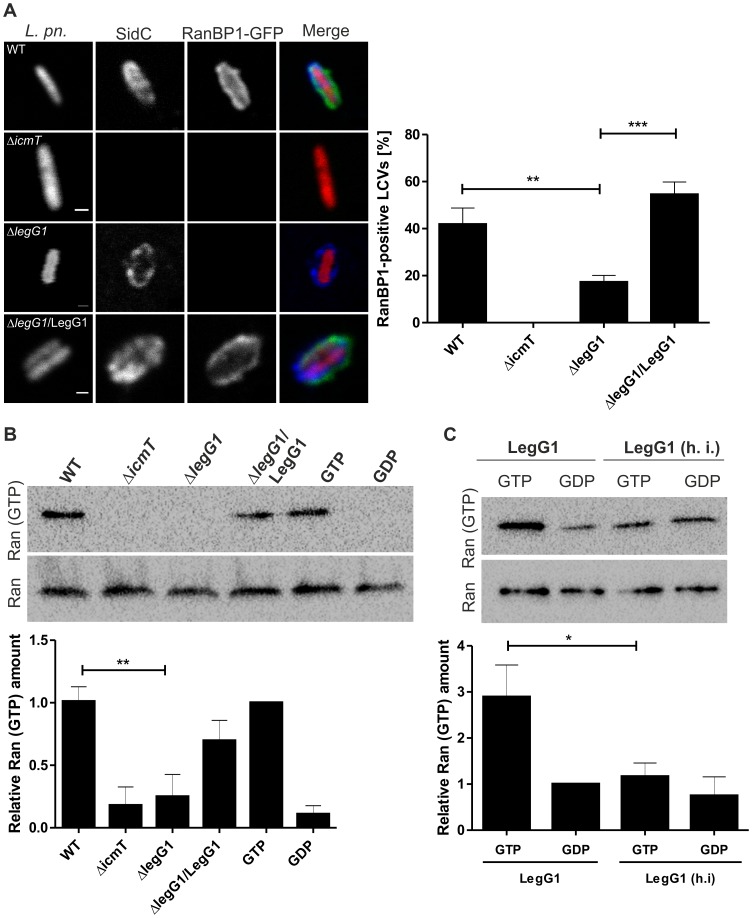
*L. pneumophila* wild-type but not Δ*legG1* activates Ran on LCVs. (**A**) LegG1 promotes RanBP1 accumulation on LCVs. *D. discoideum* producing RanBP1-GFP (green) was infected (MOI 50, 1 h) with DsRed-producing *L. pneumophila* wild-type, Δ*icmT* or Δ*legG1* harboring pCR077 (red), or with Δ*legG1*/pER5 (M45-LegG1) and immuno-stained for SidC (blue). The percentage of RanBP1-GFP-positive LCVs (n = 100/strain, 5 independent experiments) was scored in lysates of infected cells (**, *p*<0.01; ***, *p*<0.001). (**B**) Production of Ran(GTP) in infected macrophages. RAW264.7 macrophages were infected (MOI 25, 1 h) with *L. pneumophila* wild-type, Δ*icmT* or Δ*legG1* harboring pCR033 (vector) or with Δ*legG1*/pSU19 (M45-LegG1). The infected macrophages were lysed, activated Ran was immuno-precipitated with an antibody specifically recognizing Ran(GTP) and visualized by Western blot using an anti-Ran antibody. Lysates of uninfected cells incubated with GTP or GDP in presence of EDTA were used as positive or negative controls for endogenous GEF activity. Loading control: Western blot of Ran in samples before immuno-precipitation. (**C**) Production of Ran(GTP) in cell lysates. A549 epithelial cells were lysed and incubated with purified His_6_-LegG1 (native or heat-inactivated, h. i.) in presence of excess GTP (100 µM) or GDP (1 mM). Activated Ran was immuno-precipitated with an antibody specifically recognizing Ran(GTP) and visualized by Western blot using an anti-Ran antibody. Loading control: Western blot of Ran in samples before immuno-precipitation. The relative amount of Ran(GTP) was determined by densitometry; means and standard deviations of 4 (B, C) independent experiments are shown (*, *p*<0.05; **, *p*<0.01).

To analyze the function of LegG1 genetically, an *L. pneumophila* strain lacking *legG1* (Δ*legG1*, Table S1) was constructed by deleting the gene from the chromosome by double homologous recombination. LCVs harboring Δ*legG1* mutant bacteria stained for the GTPase Ran faintly but to the same extent as wild-type *L. pneumophila* (Figure 1A), suggesting that LegG1 is dispensable for the recruitment of the small GTPase to the pathogen vacuole. In contrast, however, significantly fewer LCVs containing Δ*legG1* acquired detectable levels of the Ran effector RanBP1 compared to wild-type *L. pneumophila* (Figure 2A). Less than 50% of LCVs containing Δ*legG1* stained positive for RanBP1 compared to wild-type LCVs, and the phenotype of the Δ*legG1* mutant strain was fully complemented by expressing plasmid-encoded M45-*legG1*. This finding indicates that LegG1 promotes the accumulation of RanBP1 on LCVs and thus activates Ran on LCV membranes.

To determine more directly whether *L. pneumophila* activates Ran and whether LegG1 plays a role in this process, we assayed the production of activated Ran in pulldown experiments using an antibody specifically recognizing Ran(GTP), as detailed in the Materials and Methods section. To this end, RAW264.7 macrophages were infected with *L. pneumophila* wild-type, Δ*icmT* or Δ*legG1*, or with Δ*legG1* expressing M45-*legG1*. The infected macrophages were lysed and Ran(GTP) was immuno-precipitated and visualized by Western blot using an anti-Ran antibody. Ran(GTP) was detected following infection with *L. pneumophila* wild-type but not with Δ*icmT* or Δ*legG1*, and the phenotype of the Δ*legG1* mutant strain was complemented by providing *legG1* on a plasmid (Figure 2B). Therefore, LegG1 is required to catalyze the activation of Ran by *L. pneumophila*.

A similar pulldown experiment was performed by adding purified His_6_-LegG1 to lysates of A549 cells (Figure 2C). Under these conditions, Ran(GTP) was produced in cell lysates upon addition of native (but not heat-inactivated) LegG1 in presence of GTP (but not GDP). Moreover, purified LegG1-His_6_ but not the mutant protein LegG1_N223A_-His_6_ also activated Ran in cell lysates (Fig. S3C).

Using purified N- or C-terminally His-tagged LegG1 fusion constructs, we also attempted to directly measure Ran GEF activity *in vitro* with fluorescent mantGDP (2′/3′-O-(N-methyl-anthraniloyl)-guanosine-5′-diphosphate). As a positive control, the human Ran GEF RCC1 significantly stimulated mantGDP-release from Ran(mantGDP) in the presence of excess GTP as indicated by a rapid decrease in mantGDP-fluorescence. However, under various conditions tested the purified LegG1 fusion constructs did not show Ran GEF activity *in vitro* even at elevated concentrations (Fig. S3; data not shown). In summary, these results demonstrate that *L. pneumophila* activates Ran in infected protozoan and mammalian host cells by means of a translocated effector and that LegG1 is required to activate Ran.

### LegG1 promotes intracellular replication of *L. pneumophila*

**Figure 3:**
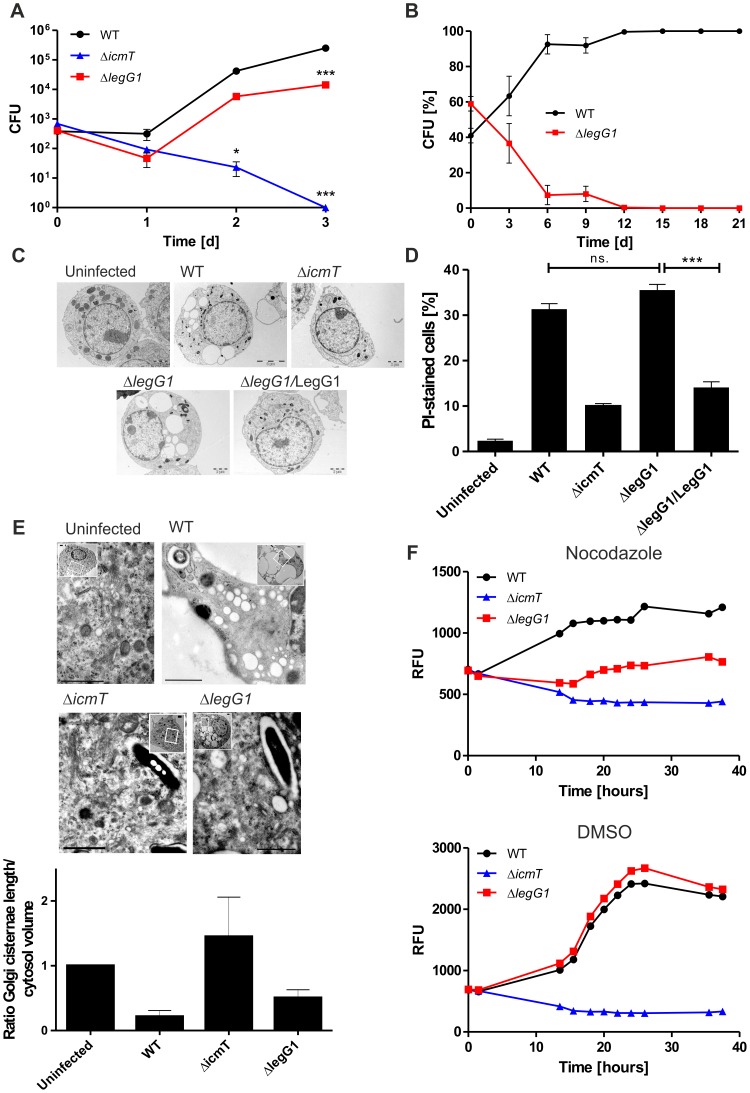
LegG1 promotes intracellular replication of *L. pneumophila*. (**A**) Intracellular replication in RAW264.7 macrophages infected (MOI 0.1) with *L. pneumophila* wild-type strain JR32, Δ*icmT* or Δ*legG1*. The infected cells were lysed and CFU were determined. Data shows means and standard deviations of triplicates and is representative of three independent experiments (*, *p*<0.05; ***, *p*<0.001). (**B**) For competition assays *A. castellanii* amoebae were co-infected with wild-type *L. pneumophila* and the Δ*legG1* mutant strain at a 1∶1 ratio (MOI of 0.01 each) and grown at 37°C during 21 d. Every third day the supernatant and lysed amoebae were diluted 1∶5000, fresh amoebae were infected, and CFU determined on CYE agar plates containing kanamycin or not. The data shown are means and standard deviations of triplicates and representative of 3 independent experiments. For toxicity assays RAW264.7 macrophages were infected (MOI 10, 4 h) with *L. pneumophila* wild-type, Δ*icmT*, or Δ*legG1* harboring pCR033, or with Δ*legG1*/pSU19 (M45-LegG1), and (**C**) analyzed by transmission electron microscopy (TEM) after embedding the sample in epoxy resin, or (**D**) analyzed by flow cytometry after detaching the cells by scraping and staining with the membrane integrity marker propidium iodide (1 µg/ml). (**E**) For TEM analysis of Golgi stacks, RAW264.7 macrophages were infected with *L. pneumophila* wild-type, Δ*icmT* or Δ*legG1* (MOI 10) and embedded in epoxy resin. The total length of Golgi cisternae and the ratio of the total number of Golgi cisternae relative to the total cytoplasmic area were quantified by stereology in thin sections. Bars, 1 µm. (**F**) Single round replication assay in RAW264.7 macrophages infected (MOI 20) with GFP-producing *L. pneumophila* wild-type, Δ*icmT* or Δ*legG1* harboring pNT28, in presence or absence of 10 µM nocodazole. Means and standard deviations of 3 samples per strain, each analyzed in triplicate, from a single experiment is shown. Data are representative of 3 independent experiments.

*L. pneumophila* lacking *legG1* grew in broth at the same rate as the isogenic wild-type strain (data not shown). Yet, the Δ*legG1* strain was slightly impaired for intracellular replication in RAW264.7 macrophages (Figure 3A) and *Acanthamoeba castellanii* (Figure S4A), but not in *D. discoideum* (Figure S4B). Upon co-infection of Δ*legG1* with wild-type *L. pneumophila* at a 1∶1 ratio, the mutant strain was efficiently out-competed by wild-type bacteria and eradicated within 6 days (Figure 3B). Thus the Ran activator LegG1 is essential for competition against wild-type *L. pneumophila* upon co-infection of amoebae. In contrast, the Rab1 GEF SidM was not required for competition under the same conditions, since a mutant strain lacking *sidM* did not show a competition defect (Figure S5).

The Δ*legG1* mutant strain was as cytotoxic for RAW264.7 macrophages as wild-type bacteria, since macrophages infected with either strain were vacuolized to the same extent (Figure 3C), and the percentage of cells permeable for propidium iodide was similar (Figure 3D). However, the overproduction of LegG1 reduced cytotoxicity significantly. The reduction of cytotoxicity was specific for LegG1, as overproduction of other effector proteins such as SidC or SidM significantly increased cytotoxicity (Figure S6A). Cytotoxicity reduction by LegG1 did not seem to be due to an impairment of type IV secretion, since translocation of the Icm/Dot substrate SidC was not affected (Figure S6B). Furthermore, *L. pneumophila* lacking or overexpressing *legG1* was taken up with the same efficiency as wild-type bacteria by amoebae (Figure S7). The uptake of *L. pneumophila* is promoted by the Icm/Dot T4SS [40], and therefore, this finding also indicates that the overproduction of LegG1 does not simply obstruct the T4SS.

To test further effects of LegG1 on host cells, we assessed the fragmentation of the Golgi apparatus by *L. pneumophila* by using transmission electron microscopy (Fig. 3E). Upon infection of RAW264.7 macrophages with wild-type *L. pneumophila* the Golgi cisternae were almost completely disrupted, while in cells infected with a Δ*icmT* mutant strain the Golgi was preserved to an extent similar to uninfected cells. In macrophages infected with *L. pneumophila* Δ*legG1* the Golgi cisternae were conserved to an intermediate degree and appeared shorter than in uninfected cells.

Finally, in absence of *legG1*, the same number of LCVs accumulated the ER/LCV marker calnexin, suggesting that LegG1 does not affect the fusion of the pathogen vacuole with the ER (Figure S7C). Together, these results indicate that LegG1 is an Icm/Dot-translocated *L. pneumophila* virulence factor that localizes to the LCV membrane, is dispensable for bacterial uptake, and promotes intracellular replication in protozoan and mammalian phagocytes.

### LegG1 stabilizes microtubules in *L. pneumophila*-infected phagocytes

Ran controls a number of cellular processes, some of which involve microtubule assembly and microtubule-dependent trafficking processes [11], [13]. To study a possible role of microtubule polymerization and LegG1 for intracellular replication of *L. pneumophila*, macrophages were treated with the microtubule-depolymerizing agent nocodazole, and the fate of GFP-producing wild-type, Δ*icmT* or Δ*legG1* mutant bacteria was monitored (Figure 3F). The growth rate of wild-type *L. pneumophila* in nocodazole-treated cells was somewhat lower compared to control cells treated with DMSO only. However, the difference in the growth rate of the Δ*legG1* strain in nocodazole-treated cells compared to control cells was much more pronounced, indicating that in the absence of the Ran activator LegG1 the depolymerization of microtubules is more deleterious for intracellular bacterial growth. Δ*icmT* mutant bacteria were unable to grow and were killed to the same extent in macrophages treated with nocodazole. These results suggest that the depolymerization of microtubules by nocodazole and the absence of the Ran activator LegG1 synergistically compromise intracellular growth of *L. pneumophila*.

**Figure 4:**
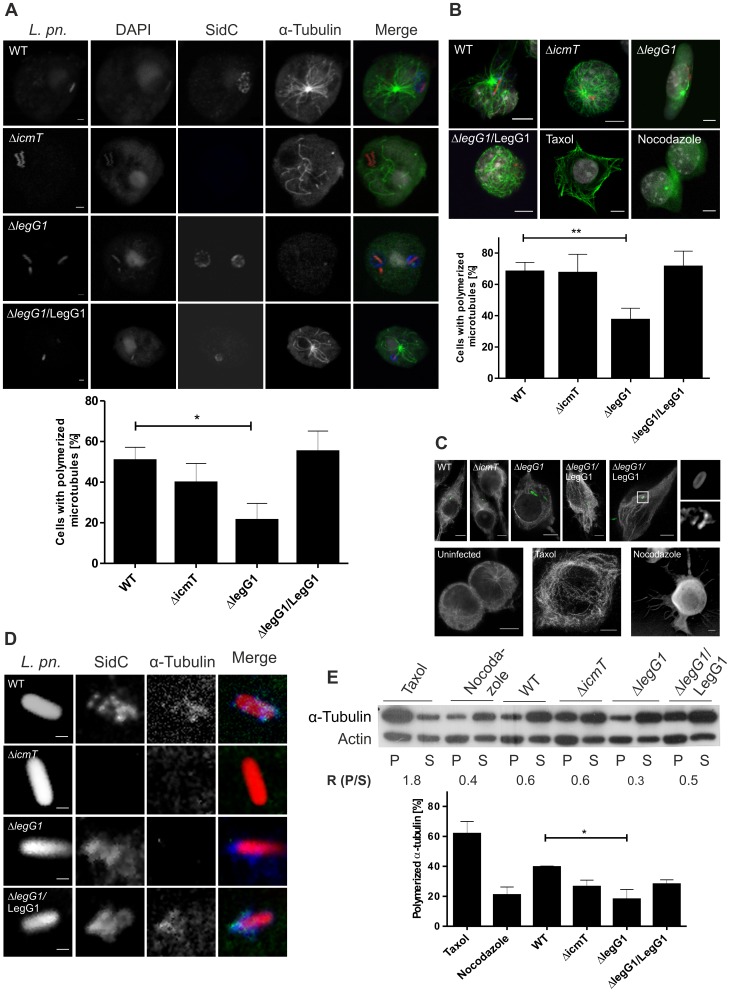
LegG1 stabilizes microtubules in *L. pneumophila*-infected phagocytes. (**A, B**) Microtubules were analyzed by confocal laser scanning fluorescence microscopy in (**A**) *D. discoideum* producing tubulin-GFP or (**B**) RAW264.7 macrophages infected (MOI 10, 2 h (amoebae), 4 h (macrophages)) with DsRed-producing *L. pneumophila* wild-type, Δ*icmT* or Δ*legG1* harboring pCR077 or with Δ*legG1*/pER5 (M45-LegG1). The macrophages were immuno-labeled for α-tubulin (green) and SidC (blue) and, as controls, treated with taxol or nocodazole (30 µM). (**C, D**) Microtubules were analyzed by STED microscopy in RAW264.7 macrophages infected (MOI 10, 4 h) with GFP-producing *L. pneumophila* wild-type, Δ*icmT* or Δ*legG1* harboring pCR076 or with Δ*legG1*/pER4 (M45-LegG1) and immuno-labeled for (**C**) α-tubulin (grey), or (**D**) α-tubulin (green) and SidC (blue). Uninfected macrophages were treated with taxol or nocodazole (30 µM) as control. Bars, 1 µm (A), 5 µm (B, C), 0.5 µm (D). (**E**) Microtubule polymerization was analyzed by anti-tubulin Western blot in RAW264.7 macrophages infected (MOI 10, 4 h) with GFP-producing *L. pneumophila* wild-type, Δ*icmT* or Δ*legG1* harboring pCR033, or with Δ*legG1*/pSU19 (M45-LegG1). As a control, uninfected macrophages were treated with taxol or nocodazole (30 µM). Homogenates of the macrophages were centrifuged (20'000×*g*, 30 min) to separate polymerized tubulin in the pellet (P) from soluble tubulin in the supernatant (S), and the amount of microtubule polymerization was assessed by α-tubulin Western blot (upper panel). Actin was used as a control. A representative experiment is shown, and the ratio of polymerized to soluble α-tubulin is indicated (R). The graph (lower panel) shows means and standard deviations of 3 independent experiments.

Next, we used confocal laser scanning fluorescence microscopy to test whether Ran activation by LegG1 promotes microtubule polymerization in *L. pneumophila*-infected phagocytes. For this purpose, a *D. discoideum* strain producing GFP-α-tubulin was infected with red fluorescent *L. pneumophila* wild-type, Δ*icmT*, Δ*legG1* or Δ*legG1* expressing M45-*legG1* (Figure 4A). In amoebae infected with wild-type *L. pneumophila* microtubules were polymerized to a greater extent compared with cells infected with the Δ*legG1* or Δ*icmT* mutant strains. More than 50% of the cells infected with Δ*legG1* showed a less dense microtubule network, and the phenotype was complemented by the *legG1* gene. In *D. discoideum* infected with the complemented strain, the majority of microtubules emanated from the centrosome and reached the cell cortex, similar to amoebae infected with wild-type *L. pneumophila*.

Similarly, in RAW264.7 macrophages infected with red fluorescent *L. pneumophila* wild-type, microtubules were polymerized to a greater extent, compared to cells infected with the Δ*legG1* mutant strain (Figure 4B). Approximately 50% of the cells infected with Δ*legG1* showed a less dense microtubule network, and again the phenotype was complemented upon providing the *legG1* gene on a plasmid. In macrophages infected with Δ*icmT*, microtubules were polymerized to a similar extent as in cells infected with wild-type *L. pneumophila*. Uninfected macrophages treated with taxol or nocodazole served as controls for microtubule polymerization or depolymerization, respectively.

The effect of the above *L. pneumophila* strains on microtubule polymerization in macrophages was further analyzed by stimulated emission depletion (STED) microscopy. This super-resolution immuno-fluorescence microscopy analysis confirmed that microtubules were polymerized to a greater extent in macrophages infected with green-fluorescent wild-type *L. pneumophila* or Δ*icmT*, compared to cells infected with Δ*legG1*, and the phenotype was complemented by providing the *legG1* gene on a plasmid (Figure 4C). Moreover, a high magnification inspection of the microtubule network in the vicinity of LCVs revealed an α-tubulin accumulation on 11% or 49% of LCVs harboring either wild-type *L. pneumophila* or the complemented Δ*legG1* strain, but not on LCVs harboring Δ*icmT* or Δ*legG1* mutant bacteria (Figure 4C, inset; Figure 4D).

The amount of polymerized or non-polymerized microtubules in *L. pneumophila*-infected macrophages was also assessed by Western blot using an anti-tubulin antibody (Figure 4E). Lysates of infected macrophages were subjected to a low and a high speed centrifugation step, and the quantity of microtubules in the pellet and the supernatant was compared. This approach revealed that macrophages infected with *L. pneumophila* lacking *legG1* contained smaller amounts of polymerized microtubules compared to cells infected with wild-type bacteria, and the phenotype was complemented by overexpression of *legG1*. In summary, different approaches revealed that *L. pneumophila* promotes microtubule polymerization in amoebae and macrophages in a LegG1-dependent manner to efficiently replicate intracellularly.

### Microbial microinjection of LegG1 promotes microtubule polymerization

**Figure 5:**
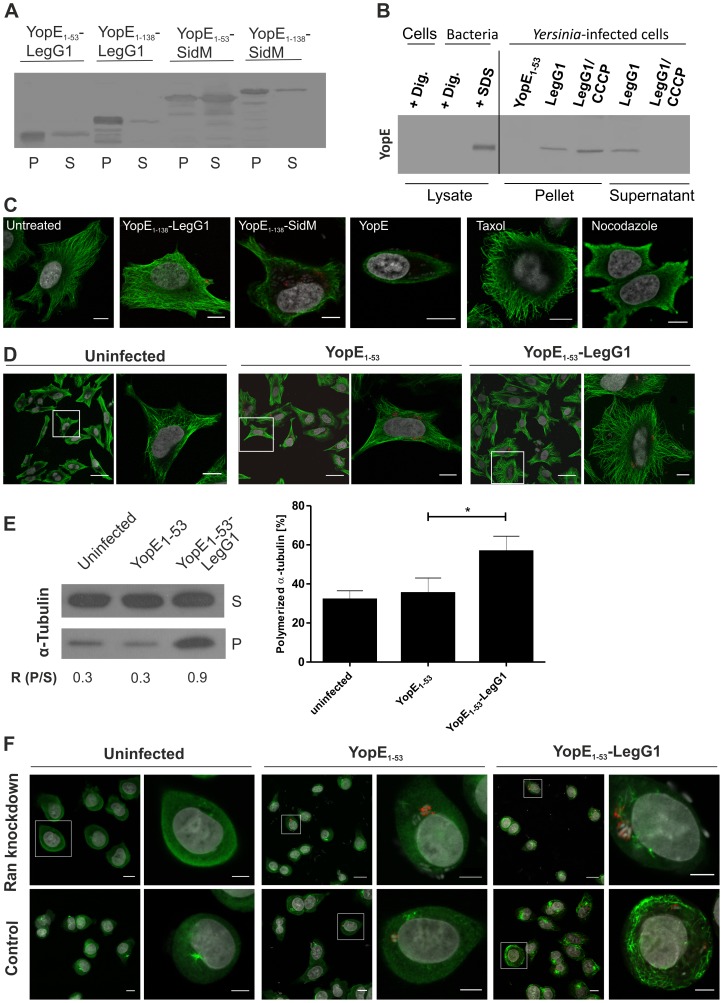
Microbial microinjection of LegG1 promotes microtubule polymerization. (**A**) *Yersinia enterocolitica* strain WA (pT3SS), encoding the Ysc T3SS but lacking type III-secreted effectors, produced and secreted fusion proteins of YopE_1–53_ or YopE_1–138_ with the *Legionella* Ran activator LegG1 or the Rab1 GEF SidM. The proteins in the pellet (P) or supernatant (S) were precipitated by chloroform/methanol treatment and visualized by Western blot using an antibody against YopE. (**B**) HeLa cells were infected (MOI 10, 2 h) with *Y. enterocolitica* WA (pT3SS) producing YopE_1–53_-LegG1, washed several times and lysed with 1% digitonin. After centrifugation, proteins in the pellet (intact bacteria, debris) and in the supernatant (translocated bacterial/soluble host proteins) were precipitated, and the fusion protein was visualized by Western blot using an anti-YopE antibody. Controls: HeLa cells alone, bacteria treated with 1% digitonin, 1% SDS or with the T3SS inhibitor CCCP (50 µM). (**C**) Fluorescence microscopy of YopE_1–138_-LegG1 translocation into HeLa cells infected (MOI 10, 2 h) with *Y. enterocolitica* WA (pT3SS) producing YopE_1–138_-LegG1, YopE_1–138_-SidM or YopE. Controls: uninfected cells or cells treated with 30 µM taxol or nocodazole. The cells were immuno-stained for α-tubulin and YopE, and nuclei were labeled with DAPI. Bars, 10 µm. (**D**) Fluorescence microscopy of YopE_1–53_-LegG1 translocation into HeLa cells treated with nocodazole (1 µM, 1 h) and infected (MOI 10, 2 h) with *Y. enterocolitica* WA (pT3SS) producing YopE_1–53_ or YopE_1–53_-LegG1. The cells were immuno-stained for α-tubulin (green) and YopE (red), nuclei were labeled with DAPI (grey). Bars, 40 µm or 10 µm (insets). (**E**) Western blot of YopE_1–53_-LegG1 translocation into HeLa cells treated with nocodazole (1 µM, 1 h) and infected (MOI 10, 2 h) with *Y. enterocolitica* WA (pT3SS) producing YopE_1–53_ or YopE_1–53_-LegG1. The soluble microtubule fraction in the cell supernatant (S) and the pellet (P) was analyzed with an anti-α-tubulin antibody; ratio of polymerized to soluble α-tubulin (R). (**F**) Fluorescence microscopy of A549 cells treated or not with siRNA against Ran and infected (MOI 10, 2 h) with *Y. enterocolitica* WA (pT3SS) producing YopE_1–53_ or YopE_1–53_-LegG1. The cells were immuno-stained for α-tubulin (green) and YopE (red), and nuclei were labeled with DAPI (grey). Bars, 10 µm or 5 µm (insets). The data shown is representative of 3 independent experiments (A–F).

As an alternative approach to analyze the effect of a single effector protein, LegG1, on host cells, we delivered the effector into host cells by microbial microinjection using the *Yersinia enterocolitica* strain WA (pT3SS). This *Yersinia* “toolbox” strain produces the Ysc type III secretion system (T3SS), yet lacks all endogenous T3SS effectors [41], [42]. N-terminal fragments of the *Y. enterocolitica* RhoG/Rac1 GAP YopE (YopE_1–53_, YopE_1–138_) are not cytotoxic but mediate secretion and translocation of hybrid proteins through the T3SS. Fusions of YopE_1–53_ or YopE_1–138_ with LegG1 or SidM were produced and secreted into the bacterial supernatant *via* the T3SS upon calcium depletion with 5 mM EGTA (Figure 5A). Moreover, dependent on the T3SS and sensitive to the protonophore CCCP (carbonyl cyanide *m*-chlorophenyl-hydrazone) heterologously produced YopE_1–53_-LegG1 was translocated into HeLa cells by *Y. enterocolitica* WA (pT3SS) (Figure 5B).

To analyze the translocation of heterologously produced effector fusion proteins, HeLa cells were infected for 2 h with *Y. enterocolitica* WA (pT3SS) producing YopE_1–138_-LegG1, YopE_1–138_-SidM or YopE, and the morphology and microtubules were analyzed by immuno-fluorescence microscopy (Figure 5C). Uninfected cells or cells infected with WA (pT3SS) producing YopE_1–138_-LegG1 showed a similar morphology and microtubule network. In contrast, cells infected with WA (pT3SS) producing YopE_1–138_-SidM or full length *Y. enterocolitica* YopE, a RhoG/Rac1 GAP [43], rounded up and the microtubules disintegrated. Uninfected HeLa cells treated with 30 µM taxol or nocodazole served as controls.

In order to visualize more subtle effects of LegG1 on the microtubule network, HeLa cells were pretreated for 1 h with 1 µM nocodazole prior to infection for 2 h with *Y. enterocolitica* WA (pT3SS) producing YopE_1–53_ or YopE_1–53_-LegG1 (Figure 5D). Immuno-fluorescence microscopy indicated that under these conditions, cells infected with *Y. enterocolitica* producing YopE_1–53_-LegG1 contained a denser microtubule network, compared with cells infected with bacteria producing YopE_1–53_ or uninfected cells. Throughout the cell body, the injection of YopE_1–53_-LegG1 caused the formation of a larger number of microtubule bundles, which emanated from the peri-nuclear region and radiated towards the cell cortex.

Microtubule polymerization triggered by *Y. enterocolitica* WA (pT3SS) producing YopE_1–53_ or YopE_1–53_-LegG1 was also quantified by Western blot using an anti-α-tubulin antibody (Figure 5E). This approach confirmed that LegG1 injected into HeLa cells significantly increased the amount of insoluble tubulin in the pellet and thus caused microtubule polymerization. Taken together, these results indicated that heterologously produced LegG1 delivered into eukaryotic cells via microbial microinjection promotes polymerization of microtubules.

Finally, to assess whether LegG1 requires the Ran GTPase to exert its effect on microtubule polymerization, A549 cells (Fig. S1) were treated with siRNA oligonucleotides silencing Ran for 2 days, followed by 1 µM nocodazole for 1 h and infection with *Y. enterocolitica* WA (pT3SS) producing YopE_1–53_ or YopE_1–53_-LegG1 for 2 h (Figure 5F). Under these conditions, LegG1 triggered microtubule condensation only in cells treated with AllStars negative control siRNA, but not in cells depleted for Ran. While 83% of the control cells infected with *Y. enterocolitica* producing YopE_1–53_-LegG1 contained a dense tubulin network, only 45% of the Ran-depleted cells did so. This experiment indicates that Ran is essential for LegG1 to promote microtubule polymerization.

### LCVs harboring *L. pneumophila* Δ*legG1* are stalled

**Figure 6:**
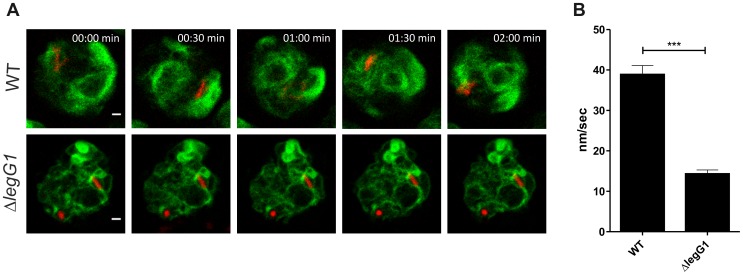
LCVs harboring *L. pneumophila* Δ*legG1* show impaired motility. (**A**) Real-time fluorescence microscopy of LCV motility in *D. discoideum* producing calnexin-GFP and infected (MOI 10, 2 h) with DsRed-producing *L. pneumophila* wild-type or Δ*legG1* mutant bacteria harboring pSW001. Two hours post infection, trafficking of LCVs was recorded by laser confocal scanning microscopy for 5 min with images taken every 15 s. Bars, 1 µm. (**B**) The velocity of LCVs was quantified by tracking the migration distance of LCVs over time (n = 50/strain; ***, *p*<0.001).

Shortly after formation, LCVs rapidly move within *D. discoideum* cells, and the pathogen vacuoles are transported along microtubules [6]. The dynamics of LCVs harboring either *L. pneumophila* wild-type (Movie S1) or Δ*legG1* mutant bacteria (Movie S2) was assessed by real-time confocal laser scanning fluorescence microscopy using calnexin-GFP-producing *D. discoideum* and DsRed-producing *L. pneumophila*. Two hours post infection the LCV motility was recorded for 5 min with images taken every 15 s (Figure 6A). While LCVs harboring wild-type *L. pneumophila* were very motile and rapidly moved along microtubules, LCVs harboring Δ*legG1* mutant bacteria were drastically slowed down and hardly moved. The velocity of LCVs was quantified by tracking the migration distance of LCVs over time. These experiments revealed that LCVs harboring wild-type *L. pneumophila* moved with a speed of about 40 nm/s within the *D. discoideum* cells, while LCVs harboring Δ*legG1* mutant bacteria moved with a 3–4 times lower speed and were almost stalled (Figure 6B).

## Discussion

In this work, we demonstrate that the small GTPase Ran, its effector RanBP1 and the Icm/Dot substrate LegG1 localize to LCVs and promote intracellular replication of *L. pneumophila*. Moreover, the *legG1* gene is required for Ran activation in infected macrophages, and purified LegG1 protein functions as a Ran activator. Finally, activation of Ran on LCVs promotes microtubule stabilization, LCV motility and intracellular growth of *L. pneumophila*. LegG1 represents the first prokaryotic Ran activator characterized. LegG1 may activate Ran either directly or indirectly. Direct activation might occur through GEF activity, similar to the activity of the eukaryotic Ran GEFs RCC1 and RanBP10. However, in a nucleotide exchange assay containing purified human Ran loaded with fluorescent mantGDP, excess GTP and His-tagged LegG1, the bacterial effector did not show GEF activity *in vitro* (Figure S3). Under the same conditions purified RCC1 efficiently catalyzed nucleotide exchange. Importantly, LegG1 harbors three RCC1 domains, while the human RCC1 GEF harbors as many as seven of these domains forming a seven-bladed propeller structure (Figure S8). Given the structural differences between LegG1 and RCC1, the former might promote the activation of Ran not by GEF activity. Possibly, LegG1 stabilizes activated Ran by binding to Ran(GTP), or the bacterial effector functions indirectly as a Ran GAP inhibitor, thus preventing the inactivation of Ran. Finally, the level of Ran(GTP) can also be modulated by nucleotide release proteins such as Mog1 [44], which might be targeted by LegG1.

The Ran GEF RCC1 is chromatin-bound and nuclear in interphase cells, or chromosome-associated in mitotic or post-mitotic cells. Regardless of the cell cycle phase, RCC1 produces a gradient of activated Ran originating from cellular DNA [10], [13]. In contrast, the cytoplasmic Ran GEF RanBP10 has been shown to directly bind tubulin and activate Ran [15], and therefore might provide a scaffold for cytosolic Ran activation and microtubule polymerization. Analogously to RanBP10, LegG1 localizes to the cytosol by accumulating on the cytosolic face of the LCV in *L. pneumophila*-infected cells (Figure 1C). Furthermore, LegG1 co-localizes with (but does not disrupt) the Golgi apparatus upon ectopic production in mammalian cells [37], [38]. Thus, LegG1 likely regulates in a spatiotemporal manner the production of a Ran(GTP) gradient originating from (a) subcellular membrane-bound compartment(s). While LegG1 localizes to the LCV membrane, the effector might not only act as a Ran activator *in cis* (on LCVs) but also *in trans* (in a distance from LCVs) to promote the formation of a replication-permissive compartment and/or to affect other cellular processes regulated by Ran. In this context it is interesting to note that the *L. pneumophila* Icm/Dot substrate RomA (Regulator of methylation A) is targeted to the host cell nucleus, where the methyltransferase modifies chromatin and gene expression by producing a novel histone mark [45]. It will be interesting to assess, whether by activating Ran LegG1 regulates nucleo-cytoplasmic transport, and thereby, the activity of RomA.

Membrane localization of LegG1 is modulated by prenylation (likely geranyl-geranylation) of a C-terminal CAAX motif [38], implying that the bacterial Ran activator subverts essential host lipidation machinery to direct its subcellular localization. Prenylation might contribute to the specific subcellular distribution among different membranous compartments and thus play an important role for the function of LegG1. Instead of the prenylation machinery, the *L. pneumophila* Rab1 GEF SidM exploits the PI lipid metabolism of the host cell and specifically binds PtdIns(4)*P* to anchor to the LCV membrane [32], [46]. Thus, *L. pneumophila* employs two different but analogous strategies to exploit host lipids as membrane anchors for bacterial GEF effector proteins targeting distinct eukaryotic small GTPases.

Ran activation in mitotic or post-mitotic cells controls the assembly of microtubule spindles and the reconstitution of the nuclear membrane envelope, a process which requires vesicle trafficking, recruitment and fusion [10], [13]. Similarly, Ran activation by translocated LegG1 positively regulates microtubule polymerization (Figure 4, 5), and LCV motility (Figure 6). Microtubule-dependent motility of LCVs might reposition the pathogen vacuole in the infected phagocyte and serve to localize the vacuole in the vicinity of interacting compartments such as the ER. Alternatively or in addition, LegG1-dependent microtubule polymerization might promote vesicle trafficking processes in a distance from the pathogen vacuole, in order to promote fusion and fission events of vesicles communicating with the vacuole. Thus, LegG1-dependent microtubule polymerization likely plays a crucial role in defining the membrane dynamics and equilibrium of LCVs. In any case, the LegG1-catalyzed microtubule dynamics are essential for *L. pneumophila* infection, as in absence of LegG1, Ran or RanBP1 intracellular bacterial growth is compromised (Figure 1B, 3A, 3B).

Ran accumulates on LCVs in an Icm/Dot-dependent manner (Figure 1A). Yet, LegG1 apparently does not affect the recruitment of Ran to LCVs, and it is unknown which protein or lipid receptor(s) on LCVs the Ran GTPase binds to. In contrast, LegG1 promotes the activation of Ran on LCVs, since in absence of *legG1* less RanBP1 accumulates on LCVs in *D. discoideum*, and Ran(GTP) production is reduced in infected macrophages (Figure 2). In amoebae or macrophages infected with the Δ*legG1* mutant strain, active Ran is still detectable. This residual Ran GEF activity might be caused by eukaryotic GEFs or by *L. pneumophila* Ran activators other than LegG1. A possible candidate is the Icm/Dot substrate PpgA (Lpg2224), which shares 16% identity and 25.4% similarity with LegG1 and is also predicted to contain RCC1 domains [37].

In order to observe effects of LegG1 on host cells without potential interference by other *L. pneumophila* effector proteins, we also performed microbial microinjection using the T3SS competent *Y. enterocolitica* “toolbox” strain WA (pT3SS), which lacks all endogenous type III-secreted effector proteins. The *L. pneumophila* T4SS substrates LegG1 and SidM were secreted and translocated into HeLa cells as N-terminal fusion proteins with either the YopE_1–53_ or the YopE_1–138_ secretion/translocation signal attached (Figure 5). Thus the folding state of these relatively small (31.2 kDa or 73.4 kDa) T4SS substrates is compatible with translocation through a T3SS. LegG1 promoted the polymerization of microtubules in HeLa cells, and therefore, the effector adopted a functional conformation in the target cell. In contrast, the 105 kDa T4SS substrate SidC was produced but neither secreted nor translocated by *Y. enterocolitica* WA (pT3SS), while its 20 kDa PtdIns(4)*P*-binding domain SidC_P4C_ [47], [48] was produced and secreted upon calcium depletion (data not shown). Thus, the *Y. enterocolitica* T3SS apparently does not transport substrates exceeding a certain size or, more likely, the 90 kDa C-terminal domain of the T4SS substrate SidC adopts a folding state in *Y. enterocolitica* that is not compatible with type III secretion. Yet, in principle microbial microinjection by the *Yersinia* “toolbox” strain is suitable to functionally deliver into host cells not only T3SS substrates but also heterologous T4SS substrates.

In summary, we document here a characterization of the first bacterial Ran activator, *L. pneumophila* LegG1. This finding paves the way for the future analysis of the signal transduction pathways activated by Ran(GTP) in *L. pneumophila*-infected phagocytes, which are implicated in microtubule polymerization, LCV motility and intracellular bacterial replication.

## Materials and Methods

### Bacteria, cells, growth conditions and infection

Bacteria and cells are listed in Table S1. *L. pneumophila* strains were grown for 3 days on CYE agar plates containing charcoal yeast extract, buffered with N-(2-acetamido)-2-amino-ethanesulfonic acid (ACES). Liquid cultures were inoculated in AYE medium at an OD_600_ of 0.1 and grown at 37°C to an OD_600_ of 3.0 (21–22 h). Chloramphenicol (Cam; 5 µg/ml) and IPTG (1 mM) were added when needed.

Murine RAW264.7, as well as human HeLa and A549 lung epithelial carcinoma cells were cultivated in RPMI 1640 medium amended with 10% heat-inactivated fetal bovine serum and 1% glutamine (all from Life Technology). *D. discoideum* strains (Table S1) were grown and transfected by electroporation as described [47], [49], and *A. castellanii* (ATCC 30234) was propagated as described [50].

The infection of phagocytes by *L. pneumophila* wild-type, Δ*icmT* or Δ*legG1* mutant strains producing GFP was analyzed as described using *A. castellanii*, *D. discoideum* or murine RAW264.7 macrophages as host cells [32], [47], [48], [50], [51]. Briefly, the phagocytes were infected with *L. pneumophila* grown for 21–22 h in AYE broth (MOI 1–50), the infection was synchronized by centrifugation (450×*g*, 10 min, RT), and the infected phagocytes were incubated at 37°C, 30°C or 25°C (*D. discoideum*) for the time indicated.

### Chromosomal deletion of *legG1* and plasmid construction

All plasmids and oligonucleotides used are listed in Table S1 or Table S2, respectively. DNA manipulations were performed according to standard protocols, and plasmids were isolated using commercially available kits from Macherey-Nagel. All PCR fragments were sequenced.

The chromosomal deletion of *legG1* was performed as described [50]: 800 bp upstream and downstream fragments of *legG1* (lpg1976) were amplified by PCR using the primer pairs oSU94/oSU95 and oSU96/oSU97, respectively, and chromosomal *L. pneumophila* DNA as a template. Both fragments were inserted by a four way ligation into a pGEM-T easy vector with a Kan^R^ cassette in-between using *Bam*HI sites and adenosine overhangs, yielding plasmid pSU1. Clones were analyzed by restriction digestion and sequencing. The Kan^R^ cassette flanked by upstream and downstream fragments was transferred into the pLAW344 suicide plasmid using *Not*I, yielding plasmid pSU2. *L. pneumophila* JR32 was transformed by electroporation with pSU2 and selected for Kan^R^/Suc^R^ and Cam^S^ colonies. Positive clones were tested by PCR, using the primers oSU94, oSU97, oKan3′ and oKan5′, and by sequencing.

Translational M45-fusion proteins of *legG1* were constructed by PCR amplification using the primer pairs: oCR158/oCR160 or oER3/oER7 and chromosomal DNA of *L. pneumophila* JR32 as a template. The fragments were cut with the appropriate restriction enzymes and inserted into pMMB207-RBS-C-M45 (pCR33), pMMB207-C-RBS-*gfp*-RBS (pCR76), pMMB207-C-RBS-DsRed-RBS (pCR77), yielding the plasmids pSU19, pER4 and pER5, respectively. The plasmids pSU17 and pSU26 (encoding RanA-GFP or RanBP1-GFP) were constructed by PCR-amplification of the corresponding genes using the oligonucleotides listed in Table S2. *D. discoideum* cDNA was used as a template, cut with *Nsi*I and inserted into pSW102 cut with the same restriction enzyme.

Plasmids encoding LegG1-His_6_ (pER2) or His_6_-LegG1 (pER3) were constructed using the primers oER4/oER6 or oCR158/oER05, respectively, and chromosomal DNA from *L. pneumophila* strain JR32 as a template. The PCR fragment was cut with *Nco*I/*Sal*I or with *Bam*HI/*Sal*I and cloned into the vector pET-28a. Plasmid pER35 encoding LegG1_N223A_-His_6_ was obtained with the QuickChange protocol using the primers oER22 and oER23, and plasmid pER2 as a template.

To construct plasmids encoding YopE_1–53_ (5.5 kDa) or YopE_1–138_ (14.8 kDa) fusion proteins produced in *Y. enterocolitica*, the *legG1* gene was amplified by PCR using the oligonucleotides oER158/oER3 and chromosomal DNA of *L. pneumophila* JR32 as template. The PCR fragment was cut with *Bam*HI/*Sal*I and cloned into pCJYE53-G3 or pCJYE138-G3 cut with the same enzymes, yielding pGP3 and pGP4, respectively. The genes *sidM*, *sidC* or *sidC*_P4C were released from plasmid pEB189, pCR6 or pHP56, respectively, by digestion with *Bam*HI/*Sal*I and cloned into pCJYE53-G3 or pCJYE138-G3 cut with the same enzymes. To liberate *gfp* from pCJYE53-G3 or pCJYE138-G3, the plasmids were cut with *Bam*HI/*Sal*I, filled in with Klenow polymerase and re-ligated.

### Translocation assay

To determine Icm/Dot-dependent translocation into host cells of LegG1, 5×10^5^/ml RAW264.7 macrophages were seeded onto 96-well plates in a final volume of 100 µl/well and incubated at 37°C overnight. The macrophages were infected with *L. pneumophila* (MOI 20, 1 h, 37°C) producing TEM β-lactamase fusion proteins (kindly provided by X. Charpentier), grown for 21 h in AYE supplemented with 0.5 mM IPTG (isopropyl-β-D-thiogalactopyranoside). 20 µl of 6-fold CCF4/AM substrate (Invitrogen) was added to each well. After 90 min incubation, the fluorescence was measured with a fluorescence plate reader (FluoStar Optima, BMG Labtech) using an excitation wave length of 410 nm, and an emission of 450 nm or 520 nm, respectively. The Icm/Dot substrate LepA served as a positive control and the cytoplasmic protein FabI as a negative control.

### Purification of His-tagged LegG1

The constructs pER2 or pER3 were transformed into *E. coli* BL21(DE3) grown aerobically in LB medium at 37°C and induced with 1 mM IPTG during exponential growth for 4 h at 30°C. Alternatively, pER2 was transformed into *E. coli* BL21-CodonPlus (DE3)-RIL and grown in 5 l LB medium at 37°C to an OD_600_ of 0.5 to 0.7 before expression was induced with 0.5 mM IPTG at 20°C overnight. Cells were harvested by centrifugation (7'000×*g*, 30 min, 4°C), suspended and homogenized in lysis buffer (50 mM Tris/HCl, pH 7.5, 10% glycerol, 10 mM β-mercaptoethanol (βME), 0.5 mM PMSF, 30 ng ml^−1^ DNase), disrupted at 10,000 psi by a French Press, and the suspension was cleared by centrifugation (11'000×*g*, 30 min, 4°C).

His_6_-tagged LegG1 was purified by affinity chromatography using nickel-nitrilotriacetic acid-agarose (Qiagen) equilibrated with buffer E (50 mM Tris/HCl, pH 8.0, 10% glycerol, 10 mM βME, 10 mM imidazole), eluted with buffer E containing 250 mM imidazole and dialyzed overnight against elution buffer lacking imidazole (4°C). Some preparations of His_6_-LegG1 were further purified by gel filtration (Superdex 75 16/600; GE Healthcare, Munich, Germany) using a buffer containing 20 mM HEPES pH 7.5, 50 mM NaCl, 1 mM MgCl_2_, and 2 mM DTE (dithioerythritol).

### Guanine nucleotide exchange assay

For the GEF assay Ran GTPase was preparatively loaded with fluorescent mantGDP (2′/3′-O-(N-methyl-anthraniloyl)-guanosine-5′-diphosphate). To this end, purified human Ran was incubated for 2 h RT in the presence of EDTA in five times molar excess over MgCl_2_ and mantGDP in five times molar excess over Ran protein. Unbound nucleotides were removed by buffer exchange using a NAP-5 column (GE Healthcare, Munich, Germany) eluting the protein with exchange buffer (20 mM HEPES pH 7.5, 50 mM NaCl, 2 mM DTE, 1 mM MgCl_2_, 1 µM mantGDP). Collected fractions containing the protein were pooled and concentrated using a Spin-X UF 500 (Corning, Munich, Germany) concentrator.

The GEF-assay was performed in 1 ml fluorescence buffer (20 mM HEPES pH 7.5, 50 mM NaCl, 2 mM MgCl_2_, 2 mM DTE, 1 µM GTP) at 25°C using a fluorescence spectrometer (Fluoromax-4, Horiba Jobin Yvon). The excitation and emission wavelength of mantGDP is 345 nm and 440 nm, respectively. The release of mantGDP was monitored via the change in mant-fluorescence after addition of recombinant LegG1 or RCC1, respectively, in the presence of 100 µM GTP. The fluorescence traces have been corrected for dilution.

### Pulldown and Western blot

Ran(GTP) was identified in cell lysates by pulldown experiments using reagents from New East Biosciences. To this end, RAW264.7 macrophages in T75 tissue culture flasks were infected (MOI 20, 1 h), lysed with lysis buffer (50 mM Tris-HCl, pH 8.0, 130 mM NaCl, 10 mM MgCl_2_, 1 mM EDTA, 1% Triton X-100) containing a protease inhibitor tablet (complete; Roche) and incubated with an anti-Ran(GTP) antibody (1∶2000, New East Biosciences) together with protein A/G-agarose beads for 2 h. The beads were then washed 4 times with lysis buffer, subjected to SDS-PAGE and analyzed by Western blot using an anti-Ran antibody (1∶1000, Abcam). As positive and negative controls, uninfected cells were treated with γ-S-GTP or GDP in presence of 20 mM EDTA before incubation with the anti-Ran(GTP) antibody. Alternatively, A549 cells were washed three times with cold PBS before lysis, incubated for 30 min at 30°C with γ-S-GTP (100 µM) or GDP (1 mM), together with purified His_6_-LegG1, heat-inactivated His_6_-LegG1, LegG-His_6_, LegG1_N223A-His_6_, or human RCC1, and Ran(GTP) was immuno-precipitated as described above.

To separate polymerized and soluble tubulin, RAW264.7 macrophages seeded in 6-well plates were infected with *L. pneumophila* strains (MOI 10, 4 h), lysed with microtubule stabilization buffer (0.1 M PIPES, pH 7.6, 2 M glycerol, 5 mM MgCl_2_, 2 mM EGTA, 0.5% Triton X-100, 4 µM taxol, protease inhibitors) and centrifuged at low speed (240×*g*, 10 min, RT) and high speed (20'000×*g*, 10 min). The protein concentration of each fraction was determined with Bradford reagent, and identical concentrations of each sample were separated by SDS-PAGE. Anti-α-tubulin (1∶6000, Abcam) and anti-actin (1∶500, Abcam) antibodies were used for Western blots.

### Uptake and cytotoxicity assays

For uptake experiments *A. castellanii*, *D. discoideum* or RAW264.7 macrophages infected in 24-well plates (MOI 50, 1 h) were washed, detached by scraping and analyzed by flow cytometry. To quantify uptake, an uptake index was defined as the product of the number of cells above the gate threshold and the fluorescence intensity of the cells. Equal fluorescence intensities of different *L. pneumophila* strains were checked by a plate reader.

To determine cytotoxicity of different *L. pneumophila* strains, 2.5×10^5^ RAW264.7 macrophages were seeded onto a 24-well plate. The cells were infected with *L. pneumophila* (MOI 10, 4 h), and a bacterial input control was plated on CYE plates. After the infection, the medium was collected and replaced with PBS. To detach the infected macrophages, the plate was shaken vigorously at 1400 rpm for one minute on an Eppendorf plate incubator. The supernatant containing detached cells was combined with the stored media and centrifuged (240×*g*, 10 min). The cells were suspended in 0.5 ml PBS containing 1 µg/µl propidium iodide (15 min, 25°C, in the dark) and quantified by flow cytometry.

### Intracellular replication and competition

To analyze intracellular replication of *L. pneumophila*, exponentially growing *D. discoideum* were washed with Sörensen phosphate buffer (2 mM Na_2_HPO_4_, 15 mM KH_2_PO_4_, pH 6.0) containing 50 µM CaCl_2_ (SorC), seeded into a 96-well plate (1×10^5^ cells/ml MB medium) and allowed to adhere for 1–2 h. RAW264.7 macrophages were seeded (1×10^5^ cells/ml RPMI medium) one day before infection. *L. pneumophila* grown for 21 h in AYE broth was diluted in MB or RPMI and used for infection (MOI 1). After centrifugation, the infected phagocytes were incubated at 30°C (*A. castellanii*) or 37°C (macrophages), a bacterial input control was plated, and samples were taken at the time points indicated (t_0_ = 10 min post infection).

Single round intracellular growth of GFP-producing *L. pneumophila* was assayed in *A. castellanii* or RAW264.7 macrophages as described [52]. Fresh medium was added to the cultures two days before the experiment. One day before the experiment, the cells were suspended and seeded into a black 96-well clear bottom plate (Perkin-Elmer) at a density of 2×10^4^ (amoebae) or 8×10^4^ (macrophages) cells/well and allowed to adhere overnight. Overnight cultures of *L. pneumophila* harboring pNT28 (GFP) were grown in AYE/Cam to an OD_600_ of 3.0 (∼2×10^9^ bacteria/ml) and diluted to 8×10^6^ bacteria/ml in LoFlo low fluorescence medium (Formedium). The cells were infected (MOI 20) with 100 µl of diluted *L. pneumophila*, centrifuged and incubated at 30°C (amoebae) or 37°C (macrophages) for 48 h or longer. Nocodazole was added directly to the infection at the concentrations indicated. The GFP fluorescence was quantified at multiple time points using a plate reader (FluoStar Optima, BMG Labtech). To correlate fluorescence readings with bacterial viability, the cells were lysed at set time points using 0.8% saponin, dilutions were plated on CYE plates, and CFU were counted.

For the competition assays, *A. castellanii* (2×10^4^ per well, 96-well plate) in Ac buffer was co-infected (MOI 0.01) each with wild-type *L. pneumophila* and the Kan-resistant mutant strain to be tested. The infected amoebae were grown for 21 days at 37°C. Every third day the supernatant was combined with amoebae lysed with 0.8% saponin, diluted 1∶1000, and fresh amoebae were infected (50 µl homogenate per 200 µl amoebae culture volume). Aliquots were plated on CYE agar plates containing Kan (10 µg/ml) or not to determine CFU.

### RNA interference

For the RNA interference experiments, A549 cells were grown in 96-well plates and treated for 2 days with a final concentration of 10 nM of the siRNA oligonucleotides indicated (Table S3). To this end, the siRNA stock (10 µM) was diluted 1∶15 in RNAse-free water, and 3 µl of diluted siRNA was added per well. Allstars siRNA (Qiagen) was used as a negative control. Subsequently, 24.25 µl RPMI medium without FCS was mixed with 0.75 µl HiPerFect transfection reagent (Qiagen), added to the well, mixed and incubated for 5–10 min at RT. In the meantime, cells were diluted in RPMI medium with 10% FCS, 175 µl of the diluted cells (2×10^4^ cells) were added on top of each siRNA-HiPerFect transfection complex and incubated for 48 h. The cells were then infected (MOI 10) with GFP-expressing *L. pneumophila* wild-type grown for 21 h, diluted in RPMI, centrifuged and incubated for 1 h. The infected cells were washed 3 times with pre-warmed medium containing 10% FCS and incubated for 24 h (well plate was kept moist with water in extra wells). To determine intracellular growth of *L. pneumophila*, GFP-fluorescence was measured using a plate reader (FluoStar Optima, BMG Labtech).

### Microbial microinjection

For microbial microinjection the *Y. enterocolitica* strain WA (pT3SS) was used [41]. This *Yersinia* “toolbox” strain harbors a mini pYV virulence plasmid encoding the Ysc T3SS and the YadA adhesin, yet lacks all endogenous type III-secreted effectors [42], [53]. The T3SS recognizes an N-terminal secretion/translocation signal of *Yersinia* effectors. In particular, N-terminal fragments of the RhoG/Rac1 GAP YopE (YopE_1–53_, YopE_1–138_) are not cytotoxic but sufficient to mediate secretion and translocation of hybrid proteins. Fusion proteins composed of YopE_1–53_ or YopE_1–138_ and *L. pneumophila* LegG1, SidM, SidC or its PtdIns(4)*P*-binding domain SidC_P4C_ were produced in *Y. enterocolitica* WA (pT3SS) and analyzed by Western blot using a polyclonal anti-YopE antibody (1∶5000; gift from J. Heesemann). The production of YopE served as a positive control.

T3SS-dependent protein secretion was triggered by calcium depletion with EGTA essentially as described [53]. Briefly, *Y. enterocolitica* cultures grown overnight in BHI broth at 27°C were diluted 1∶20 in 10 ml BHI and grown at 37°C for another 1.5 h, followed by the addition of 10 ml of a solution containing 15 mM MgCl_2_, 5 mM EGTA and 0.2% glucose for 2 h (final OD_600_ 0.6–0.7). Subsequently, the cells were centrifuged (5000×*g*, 10 min), and the pellet was washed with PBS and suspended in 500 µl SDS PAGE sample buffer. The supernatant was sterile filtered, precipitated with TCA (10% v/v, 1 h, on ice) and centrifuged (20'000×*g*, 30 min, 4°C). The resulting pellet was suspended in 3 ml cold acetone (15 min, −20°C), centrifuged (20'000×*g*, 5 min, 4°C), washed once with each 1 ml cold acetone, and suspended in 50 µl SDS PAGE sample buffer. YopE and YopE fusion proteins were visualized by Western blot using an anti-YopE antibody.

Protein translocation into HeLa cells was determined by subcellular fractionation essentially as described [53], [54]. *Y. enterocolitica* producing YopE_1–53_ or YopE_1–53_-LegG1 were grown over night in BHI at 27°C, diluted 1∶20 into fresh media, grown for another 2 h at 37°C and used to infect HeLa cells (MOI 10) seeded at a density of 2×10^7^ in T75 tissue culture flasks the day before the experiment. Alternatively, the cells were infected with *L. pneumophila* (MOI 100) as described above. Two hours post infection the HeLa cells were washed several times with PBS and lysed with 1% digitonin. This treatment selectively lysed the host cells, while the bacteria remained intact. After centrifugation, proteins in the pellet (intact bacteria, debris) and in the supernatant (translocated bacterial proteins, soluble host proteins) were precipitated with methanol/chloroform, and YopE_1–53_, YopE_1–53_-LegG1 or SidC was visualized by Western blot using anti-YopE (1∶5000) or anti-SidC (1∶1000) antibodies. As controls, HeLa cells alone were used or bacteria treated with 1% digitonin, 1% SDS or 50 µM of the protonophore CCCP, a potent inhibitor of bacterial T3SSs [55], [56].

To analyze protein translocation by fluorescence microscopy, HeLa cells in 24-well plates containing sterile cover slips were infected (MOI 10, 2 h) with *Y. enterocolitica* producing YopE_1–138_-LegG1, YopE_1–138_-SidM or YopE. Uninfected cells or cells treated with 30 µM taxol or nocodazole served as controls. Alternatively, HeLa or A549 cells were treated with 1 µM nocodazole (1 h) and left uninfected or were subsequently infected (MOI 10, 2 h) with *Y. enterocolitica* producing YopE_1–53_ or YopE_1–53_-LegG1. Where indicated A549 cells were treated with siRNA two days before infection as described above. The cells were immuno-stained for α-tubulin (1∶600; Abcam) and YopE (1∶5000), and nuclei were labeled with DAPI (1 µg/ml).

### (Real-time) fluorescence microscopy

Fluorescence microscopy of *D. discoideum* producing GFP fusion proteins was performed as described [47], [48], [51]. Briefly, exponentially growing cells were seeded on sterile coverslips in 24-well plates at 2.5×10^5^ per well in 1 ml HL5 medium and let grow over night. *L. pneumophila* cultures grown for 21–22 h in AYE liquid medium were diluted in HL5 medium and used for infection (MOI 50). The infection was synchronized by centrifugation, and the infected cells were incubated at 25°C for the time indicated and fixed on cover slips.

To obtain homogenates, infected amoebae were washed with cold SorC one hour post infection, suspended in homogenization buffer (20 mM HEPES, 250 mM sucrose, 0.5 mM EGTA, pH 7.2) [57] and lysed by seven passages through a ball homogenizer (Isobiotech) using an exclusion size of 8 µm. The homogenate was centrifuged onto coverslips coated with poly-L-lysine, fixed with 4% paraformaldehyde (PFA) for 30 min at 4°C and blocked with 1% BSA in SorC for 30 min.

The coverslips containing homogenates were incubated for 1 h at RT on parafilm with 40 µl of primary antibody diluted in blocking buffer (affinity purified rabbit anti-SidC (1∶100) [48]; anti-M45 1∶300, Genovac AG) and washed 3 times. Appropriate secondary antibodies were diluted 1∶200 in blocking buffer and incubated for 1 h at RT. Finally, the coverslips were washed and mounted using Vectashield (Vector Laboratories) supplemented with 1 µg/ml DAPI to stain DNA. The samples were viewed with a Leica TCS SP5 confocal microscope (HCX PL APO CS, objective 63×/1.4-0.60 oil; Leica Microsystems, Mannheim, Germany).

Microtubule cytoskeleton analysis was performed with RAW264.7 macrophages or *D. discoideum* infected with DsRed- or GFP-producing *L. pneumophila* (MOI 10, 4 h, 37°C). Subsequently, the infected phagocytes were washed once with Brb80, and fixed (50% Brb80, 0.1% Triton X-100, 0.5% glutaraldehyde) for 5 min. After washing with SorC, samples were blocked with 1 mg/ml sodium borohydrate in SorC for 10 min. The samples were stained with the anti-α-tubulin antibody WA3 (gift from M. Schleicher) and anti-SidC (1∶100) on parafilm for 1 h. Appropriate secondary antibodies were used at a dilution of 1∶200 (confocal microscopy) or 1∶400 (STED microscopy). 10 cells per coded sample were assessed for the degree of tubulin polymerization.

For real-time fluorescence microscopy exponentially growing *D. discoideum* amoebae producing calnexin-GFP were seeded in life-cell imaging dishes (Ibidi) in LoFlo medium containing G418 (20 µg/ml) to a total cell number of 5×10^5^ cells one day prior to the experiment. Before infection with *L. pneumophila* strains producing DsRed (MOI 10), the cells were washed with LoFlo. Ascorbic acid was added to the cells (20 mg/ml). After two hours incubation at 25°C with no centrifugation, an agar overlay was prepared and the infected cells were observed (recorded) for 5 min. Images were taken every 15 s.

### STED microscopy

Stimulated emission depletion (STED) microscopy was employed for sub-diffraction resolution fluorescence imaging on a custom-made in-house setup. The system's basic principle is described elsewhere [58]. The system used is capable of acquiring one channel with confocal and two channels with STED resolution quasi-simultaneously. For imaging GFP-producing *Legionella* and Atto 655-coupled anti-α-tubulin, we used excitation/emission wavelengths of 488±3 nm/520±14 nm and 637±5 nm/685±20 nm, respectively. The STED wavelength for Atto 655 was 750±10 nm. Beam powers for acquisition were 0.5–4.5 µW, and 7 µW for GFP and Atto 655, respectively, as measured in front of the objective. STED beam powers were 1.4 mW for Atto 655. To reduce crosstalk, pulses for various channels were separated in time by varying optical path lengths. A home-built electronic gating device transmitted detector signals occurring at the correct time to the acquisition hardware, and rejected crosstalk signals occurring at other times. Dichroics and filters were purchased from AHF, Tübingen. The supercontinuum laser source was a SC450-PP-HE system running at 1 MHz, manufactured by Fianium Ltd, Southampton. For beam-scanning, we used a YANUS IV scan head from Till Photonics, Munich. The objective was a Leica 100×/1.4.

STED image processing: For acquiring images, movies comprised of 5–20 frames were acquired at an exposure time per pixel of 20–50 µs. All frames were then added up, and a non-local means algorithm (Buades et al., 2005) was applied via a matlab script (Peyré et al., 2007) with algorithm parameters being radius of search window: 1, radius of similarity window: 10, degree of filtering: 10.

### Transmission electron microscopy

For transmission electron microscopy cells were fixed by adding 2% glutaraldehyde (GA) in 100 mM PIPES, pH 7.0, to the culture medium at a 1∶1 volume ratio. After 5 min, the supernatant was discarded, replaced with fresh 1% GA in the same PIPES buffer and incubated overnight at RT. For the subsequent epoxy resin embedding, either (i) cells were scraped, spun down and re-suspended in non-supplemented PIPES buffer, or (ii) the fixative of adherent cells was exchanged with non-supplemented PIPES buffer.

For epoxy resin embedding, the suspended or adherent cells were washed two times with 100 mM cacodylate buffer, post-fixed with 2% OsO_4_ solution containing 1.5% potassium ferricyanide for 1 hour, and stained en block with 1.5% aqueous uranyl acetate for 30 min. The suspended cells were then dehydrated using a graded ethanol series and propylene oxide, and embedded in epoxy resin (Sigma-Aldrich; St. Louis/MO, USA). The cell monolayer was dehydrated using a graded ethanol series and detached from the bottom of the culture dish by dissolving the plastic with propylene oxide followed by rapid, vigorous pipetting. The cells were then washed four times with propylene oxide to remove the remains of dissolved plastic and thereafter embedded in epoxy resin. Ultrathin sections of 60–70 nm were cut with an ultramicrotome (Leica Ultracut UCT), stained with 0.2% lead citrate (Taab; Berks, England) in 0.1 M NaOH for 20 s and examined in a Philips CM100 transmission electron microscope.

For the stereological analysis of Golgi cisternae, two whole sections from two blocks of each sample were systematically sampled at 900× magnification in order to estimate the cytoplasmic area (volume). Within these micrographs all areas containing identifiable Golgi cisternae were selected and imaged at 8.900× magnification. To analyze the micrographs, a stereological test grid with horizontal and vertical lines was used. The total volume of the cytoplasm, which represented a reference space, was estimated by counting the number of test points over the cytoplasm. In order to estimate the total length of the Golgi cisternal membrane, the number of intersections of all identifiable Golgi membrane with horizontal test lines was counted. In addition, the number of Golgi cisternae - defined as an elongated enclosed membrane profile with a length minimum twice its breadth - was counted in these images. From these values the ratios of the total number of intersections (length) of Golgi cisternal membranes to the total volume of cytoplasm (relative surface density of cisternae) was estimated, as was the relative number of cisternae per cytoplasmic volume for the different conditions. At least 80 cell profiles were analyzed for each sample, and two independent experiments were performed. Within each experiment indices of the estimated values were calculated as ratios compared to the wild-type sample.

### Supporting information

Supporting information includes eight figures (Figure S1, S2, S3, S4, S5, S6, S7, S8), three tables (Table S1, S2, S3) and two movies (Movie S1, S2) and can be found with this article online.

### Accession numbers

The GenBank (http://www.ncbi.nlm.nih.gov/Genbank) accession numbers for the proteins discussed in this paper are *L. pneumophila* Icm/Dot T4SS (Y15044), LegG1/Lpg1976 (YP_095992), SidC (AY504673), *D. discoideum* calnexin (AF073837), human Ran GTPase (CAG29343) and RanBP1 (CAG30442).

## Supporting Information

Figure S1 Effects of siRNA treatment on intracellular replication, cytotoxicity or protein depletion efficiency. (**A**) Untreated A549 lung epithelial cells immuno-stained for α-tubulin (green); nuclei were labeled with DAPI (grey). Microtubule polymerization was analyzed by immuno-fluorescence microscopy. Bars, 10 µm or 5 µm (insets). A549 cells were treated for 2 days with 10 nM of four different siRNA oligonucleotides per target (Table S3). AllStars negative control siRNA (Qiagen) served as negative control. (**B**) Intracellular replication of GFP-producing *L. pneumophila* harboring pNT28 was monitored over 2 days and quantified by fluorescence measurement. The data represent mean and standard deviation of three independent experiments. (**C**) Cytotoxicity of siRNA treated cells was assessed by adding propidium iodide (1 µg/ml, 15 min) to detached cells, followed by flow cytometry analysis. (**D**) The depletion efficiency of the siRNA treatment was assessed by Western blot using antibodies against Ran or RanBP1. Loading control: glyceraldehyde-3-phosphate dehydrogenase (GAPDH).
(TIF)

Figure S2 Icm/Dot-dependent translocation of LegG1. RAW264.7 macrophages were infected (MOI 20) with *L. pneumophila* wild-type strain JR32 or Δ*icmT* harboring pXDC61-*legG1*, pXDC61-*lepA* or pXDC61-*fabI* encoding TEM β-lactamase fusion proteins. Enzymatic activity was assayed through hydrolysis of the fluorogenic substrate CCF4/AM (emission ratio 460/530 nm).
(TIF)

Figure S3 Analysis of Ran GEF activity of LegG1 *in vitro*. (**A**) LegG1 does not show GEF activity toward Ran:mantGDP *in vitro*. Purified His_6_-LegG1 (620 nM) and RCC1 (100 nM) were sequentially added as indicated to 1 µM purified human Ran GTPase loaded with fluorescent mantGDP. The addition of His_6_-LegG1 did not stimulate mantGDP release from Ran:mantGDP in presence of excess GTP (100 µM), whereas the human Ran GEF RCC1 significantly accelerated mantGDP-GTP exchange, as indicated by a rapid exponential change in mant-fluorescence. Fluorescence has been corrected for dilution effects. (**B**) Production of Ran(GTP) in lysates of A549 cells treated with purified His_6_-LegG1 or RCC1, or (**C**) purified LegG1-His_6_ or LegG1_N223A-His_6_ in presence of excess GTP (100 µM). Activated Ran was immuno-precipitated with an antibody specifically recognizing Ran(GTP) and visualized by Western blot using an anti-Ran antibody. Loading control: Western blot of Ran in samples before immuno-precipitation.
(TIF)

Figure S4 Replication of *L. pneumophila* Δ*legG1* in amoebae. (**A**) *A. castellanii* amoebae were infected (MOI 20) with *L. pneumophila* wild-type, Δ*icmT*, or Δ*legG1* harboring pNT28 (GFP), and intracellular growth (“single round replication”) was monitored by GFP fluorescence. Representative time course from a single experiment is shown (12 samples per strain), indicating mean fluorescence and 95% confidence intervals; data are representative of at least 3 independent experiments. (**B**) *D. discoideum* was infected (MOI 1) with *L. pneumophila* wild-type strain JR32, Δ*legG1* or Δ*icmT*, and bacteria released into the supernatant were quantified by CFU.
(TIF)

Figure S5 Amoebae competition assay of *L. pneumophila* Δ*sidM. L. pneumophila* Δ*sidM* is not outcompeted by wild-type bacteria in the amoebae competition assay. *A. castellanii* was co-infected (1∶1 ratio, MOI 0.01) in 96-well plates with *L. pneumophila* wild-type and the Δ*sidM* mutant strain, and grown at 37°C for 21 d. Every third day the supernatant and lysed amoebae were diluted 1∶1000, fresh amoebae were infected (50 µl homogenate per 200 µl culture), and aliquots were plated on CYE agar plates containing kanamycin or not to determine CFU. The data shown are means and standard deviations of triplicates and representative of 3 independent experiments.
(TIF)

Figure S6 Toxicity and effector translocation of *L. pneumophila* Δ*legG1* strains overproducing SidM or SidC. (**A**) For toxicity assays RAW264.7 macrophages were infected (MOI 10, 4 h) with *L. pneumophila* wild-type harboring pCR033 (vector), pCR034 (M45-SidC) or pEB201 (M45-SidM), detached from the wells by scraping, stained with propidium iodide (1 µg/ml) and analyzed by flow cytometry. (**B**) To assay translocation efficiency, HeLa cells were infected (MOI 100, 1 h) with *L. pneumophila* wild-type, Δ*icmT* or Δ*legG1* harboring the vector pCR033, or with Δ*legG1*/pSU19 (M45-LegG1), washed several times and lysed with 1% digitonin. 25 µl lysate of uninfected or infected HeLa cells or wild-type *L. pneumophila* were separated by SDS PAGE and stained with Coomassie Brilliant Blue, or were subjected to Western blot using an anti SidC antibody to quantify the amount of translocated effector protein.
(TIF)

Figure S7 Uptake and LCV formation of *L. pneumophila* Δ*legG1. L. pneumophila* Δ*legG1* is not impaired for uptake and LCV formation. (**A**) *A. castellanii* or (**B**) *D. discoideum* was infected (MOI 20, 45 min) with GFP-producing *L. pneumophila* wild-type, Δ*legG1* or Δ*icmT* harboring pCR076, or with Δ*legG1*/pER4 (M45-LegG1), and uptake was determined by flow cytometry. (**C**) *D. discoideum* producing calnexin-GFP was infected (MOI 50, 1 h) with DsRed-producing *L. pneumophila* wild-type, Δ*legG1* or Δ*icmT* harboring pCR077, or with Δ*legG1*/pER5 (M45-LegG1). The percentage of calnexin-GFP-positive LCVs (n = 100/strain, 4 independent experiments) was scored in lysates of infected cells.
(TIF)

Figure S8 Comparison of *L. pneumophila* LegG1 with human RCC1. (**A**) Amino acid sequence of the 31.2 kDa *L. pneumophila* protein LegG1/Lpg1976 (286 amino acids). The three RCC1 domains, which are predicted by the PROSITE program (http://prosite.expasy.org/), are highlighted in red. (**B**) Schematic overview and position of RCC1 domains in *L. pneumophila* LegG1 and human RCC1 Ran GEF. (**C**) Alignment of the three RCC1 domains of LegG1 with a single RCC1 domain of RCC1. (**D**) Predicted structure of LegG1 (Phyre2; http://www.sbg.bio.ic.ac.uk/phyre2) and comparison with the X-ray crystallography structure at 1.7 Å resolution of human RCC1 forming a seven-bladed propeller (Renault *et al.* (1998) Nature 392: 97–101).
(TIF)

Movie S1 Motility of LCVs in *D. discoideum* infected with *L. pneumophila* wild-type. *D. discoideum* amoebae producing calnexin-GFP were infected (MOI 10) with *L. pneumophila* wild-type harboring pSW001 (DsRed). Two hours post infection, the trafficking of LCVs was recorded by laser confocal scanning microscopy for 5 min, and images were taken every 15 s. The speed of LCVs was determined by tracking the migration distance of LCVs over time. Bars, 1 µm.
(AVI)

Movie S2 Motility of LCVs in *D. discoideum* infected with *L. pneumophila* Δ*legG1*. LCVs harboring *L. pneumophila* Δ*legG1* show impaired motility. *D. discoideum* amoebae producing calnexin-GFP were infected (MOI 10) with *L. pneumophila* Δ*legG1* mutant bacteria harboring pSW001 (DsRed). Two hours post infection, the trafficking of LCVs was recorded by laser confocal scanning microscopy for 5 min, and images were taken every 15 s. The speed of LCVs was determined by tracking the migration distance of LCVs over time. Bars, 1 µm.
(AVI)

Table S1 Strains and plasmids.
(DOCX)

Table S2 Oligonucleotides used in this study.
(DOCX)

Table S3 Oligonucleotides used for RNA interference.
(DOCX)
