# Supplementary figures and images for: Activation of Ran GTPase by a Legionella Effector Promotes Microtubule Polymerization, Pathogen Vacuole Motility and Infection
Source: PLoS Pathog. 2013 Sep 19;9(9):e1003598. doi: 10.1371/journal.ppat.1003598 (PMC3777869; doi:10.1371/journal.ppat.1003598)

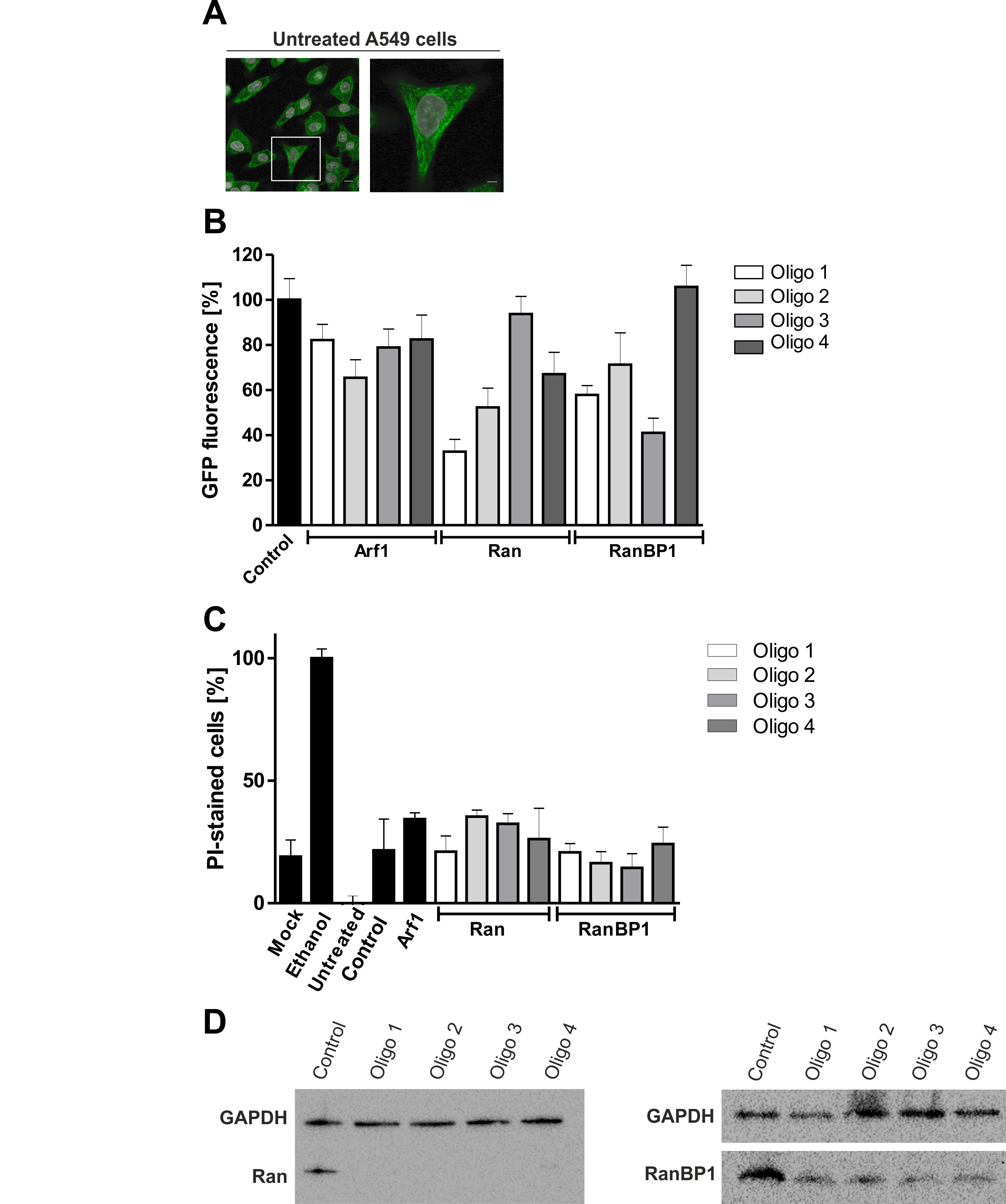

Supplement: Figure S1 — Effects of siRNA treatment on intracellular replication, cytotoxicity or protein depletion efficiency. (A) Untreated A549 lung epithelial cells immuno-stained for α-tubulin (green); nuclei were labeled with DAPI (grey). Microtubule polymerization was analyzed by immuno-fluorescence microscopy. Bars, 10 µm or 5 µm (insets). A549 cells were treated for 2 days with 10 nM of four different siRNA oligonucleotides per target (Table S3). AllStars negative control siRNA (Qiagen) served as negative control. (B) Intracellular replication of GFP-producing L. pneumophila harboring pNT28 was monitored over 2 days and quantified by fluorescence measurement. The data represent mean and standard deviation of three independent experiments. (C) Cytotoxicity of siRNA treated cells was assessed by adding propidium iodide (1 µg/ml, 15 min) to detached cells, followed by flow cytometry analysis. (D) The depletion efficiency of the siRNA treatment was assessed by Western blot using antibodies against Ran or RanBP1. Loading control: glyceraldehyde-3-phosphate dehydrogenase (GAPDH). (TIF) [file ppat.1003598.s001.tif]

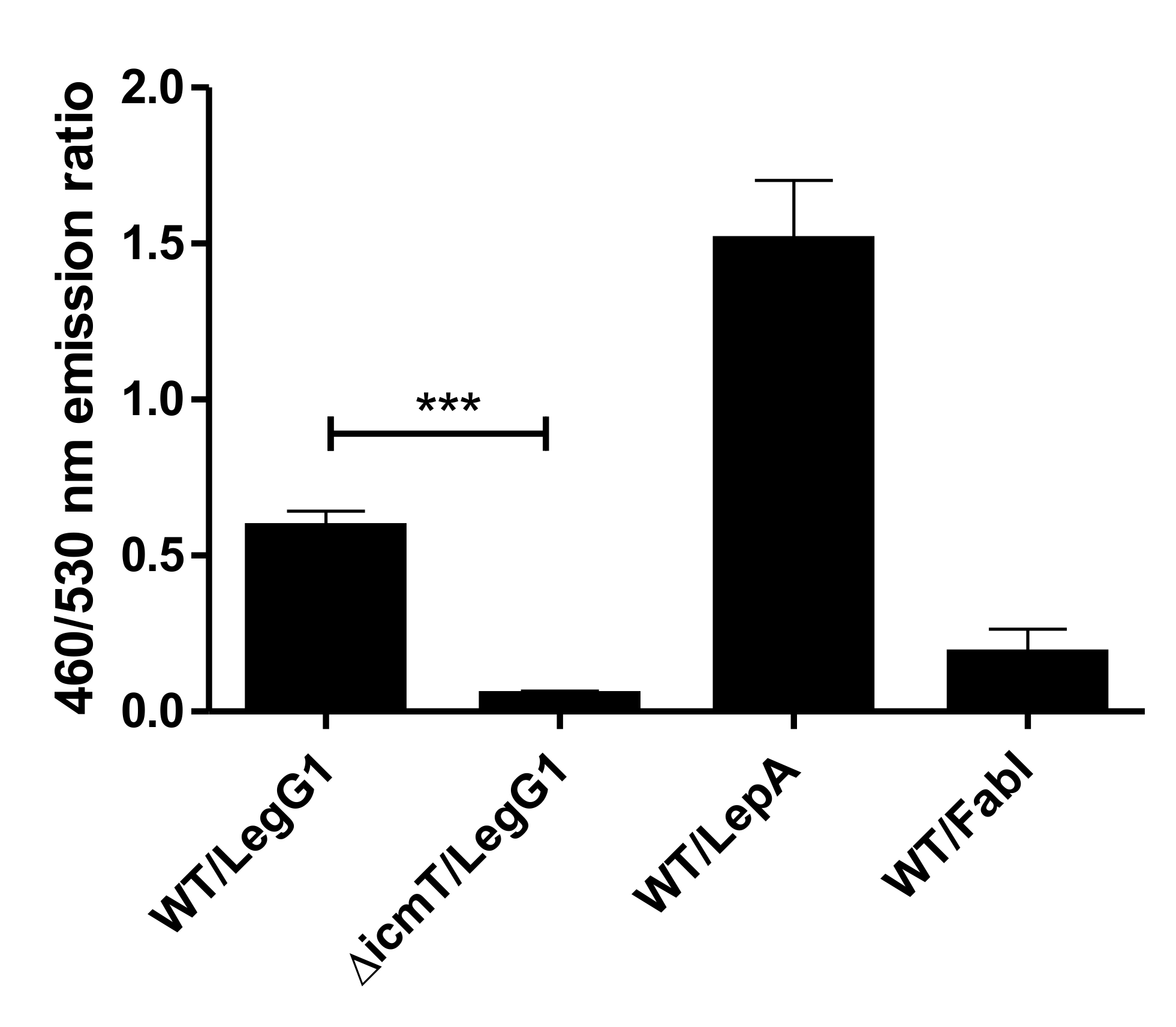

Supplement: Figure S2 — Icm/Dot-dependent translocation of LegG1. RAW264.7 macrophages were infected (MOI 20) with L. pneumophila wild-type strain JR32 or ΔicmT harboring pXDC61-legG1, pXDC61-lepA or pXDC61-fabI encoding TEM β-lactamase fusion proteins. Enzymatic activity was assayed through hydrolysis of the fluorogenic substrate CCF4/AM (emission ratio 460/530 nm). (TIF) [file ppat.1003598.s002.tif]

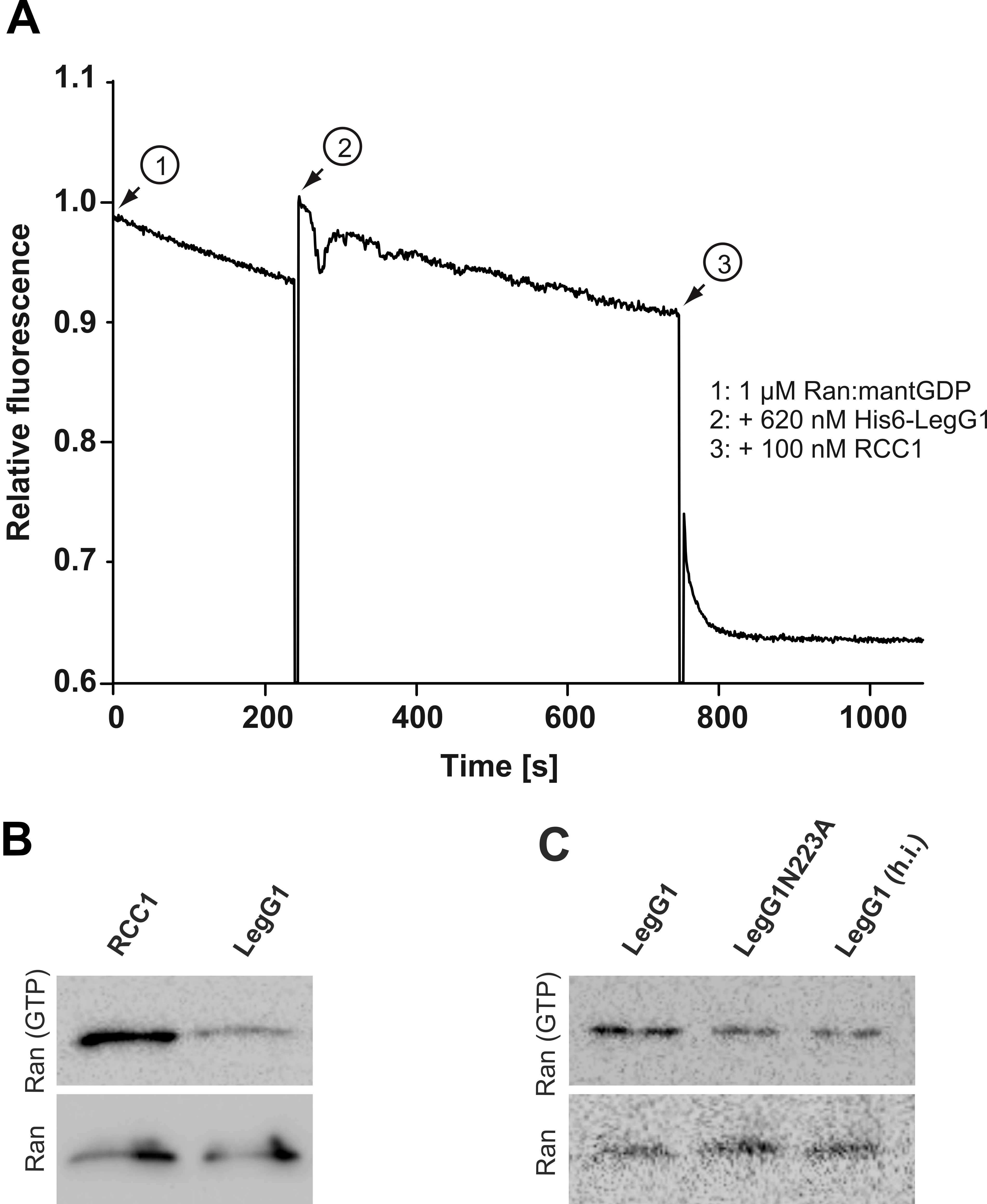

Supplement: Figure S3 — Analysis of Ran GEF activity of LegG1 in vitro. (A) LegG1 does not show GEF activity toward Ran:mantGDP in vitro. Purified His6-LegG1 (620 nM) and RCC1 (100 nM) were sequentially added as indicated to 1 µM purified human Ran GTPase loaded with fluorescent mantGDP. The addition of His6-LegG1 did not stimulate mantGDP release from Ran:mantGDP in presence of excess GTP (100 µM), whereas the human Ran GEF RCC1 significantly accelerated mantGDP-GTP exchange, as indicated by a rapid exponential change in mant-fluorescence. Fluorescence has been corrected for dilution effects. (B) Production of Ran(GTP) in lysates of A549 cells treated with purified His6-LegG1 or RCC1, or (C) purified LegG1-His6 or LegG1_N223A-His6 in presence of excess GTP (100 µM). Activated Ran was immuno-precipitated with an antibody specifically recognizing Ran(GTP) and visualized by Western blot using an anti-Ran antibody. Loading control: Western blot of Ran in samples before immuno-precipitation. (TIF) [file ppat.1003598.s003.tif]

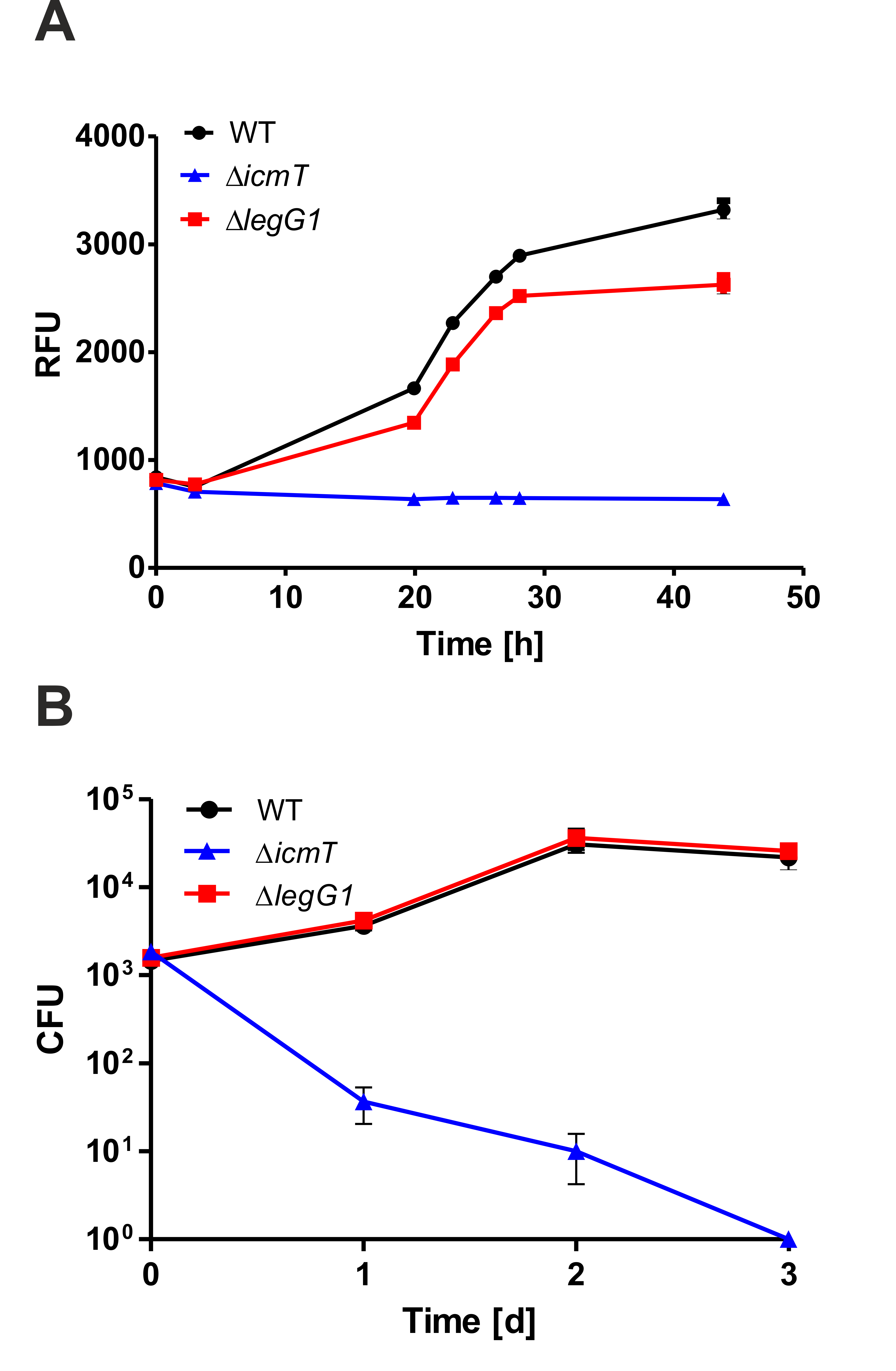

Supplement: Figure S4 — Replication of L. pneumophila ΔlegG1 in amoebae. (A) A. castellanii amoebae were infected (MOI 20) with L. pneumophila wild-type, ΔicmT, or ΔlegG1 harboring pNT28 (GFP), and intracellular growth (“single round replication”) was monitored by GFP fluorescence. Representative time course from a single experiment is shown (12 samples per strain), indicating mean fluorescence and 95% confidence intervals; data are representative of at least 3 independent experiments. (B) D. discoideum was infected (MOI 1) with L. pneumophila wild-type strain JR32, ΔlegG1 or ΔicmT, and bacteria released into the supernatant were quantified by CFU. (TIF) [file ppat.1003598.s004.tif]

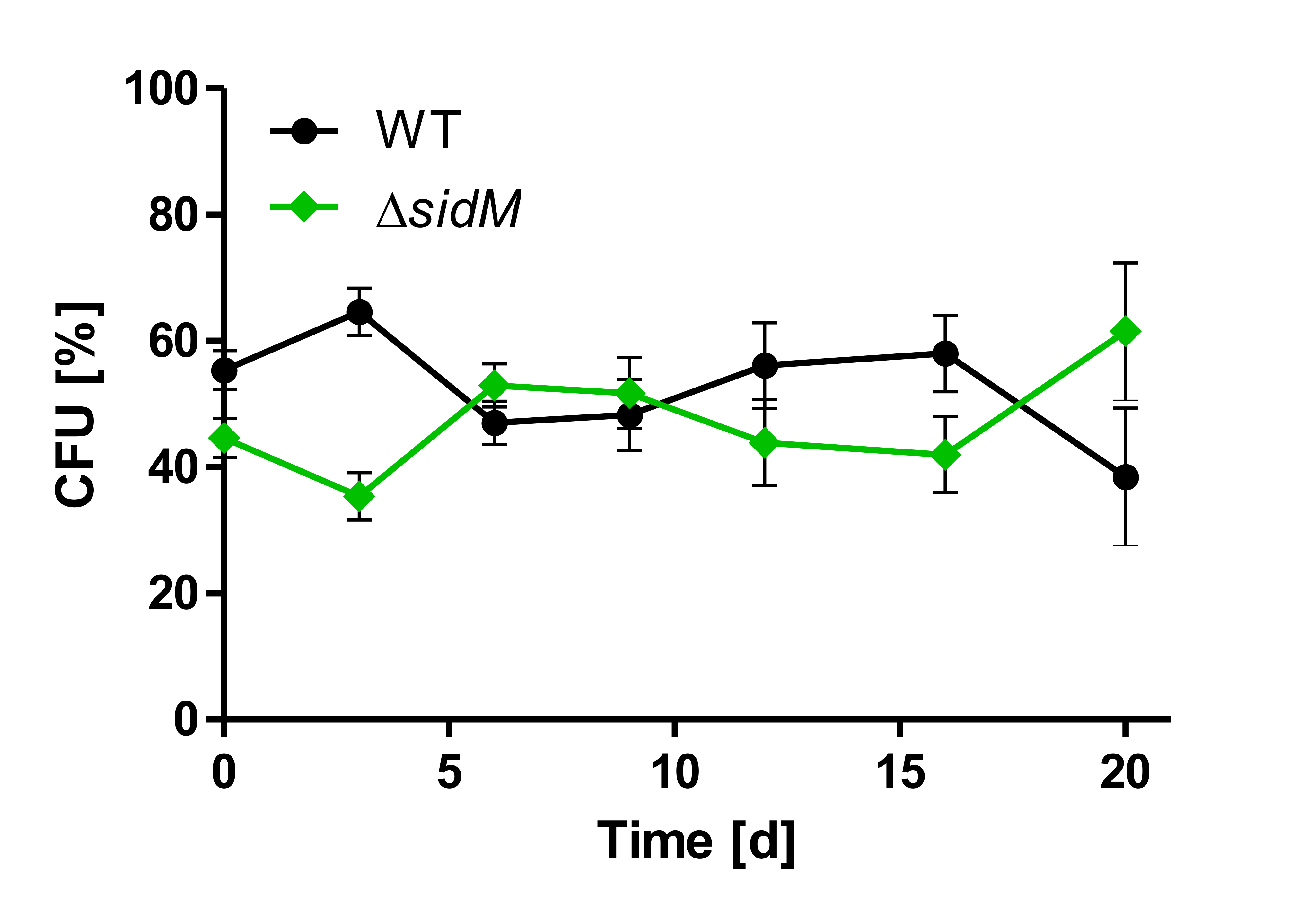

Supplement: Figure S5 — Amoebae competition assay of L. pneumophila ΔsidM. L. pneumophila ΔsidM is not outcompeted by wild-type bacteria in the amoebae competition assay. A. castellanii was co-infected (1∶1 ratio, MOI 0.01) in 96-well plates with L. pneumophila wild-type and the ΔsidM mutant strain, and grown at 37°C for 21 d. Every third day the supernatant and lysed amoebae were diluted 1∶1000, fresh amoebae were infected (50 µl homogenate per 200 µl culture), and aliquots were plated on CYE agar plates containing kanamycin or not to determine CFU. The data shown are means and standard deviations of triplicates and representative of 3 independent experiments. (TIF) [file ppat.1003598.s005.tif]

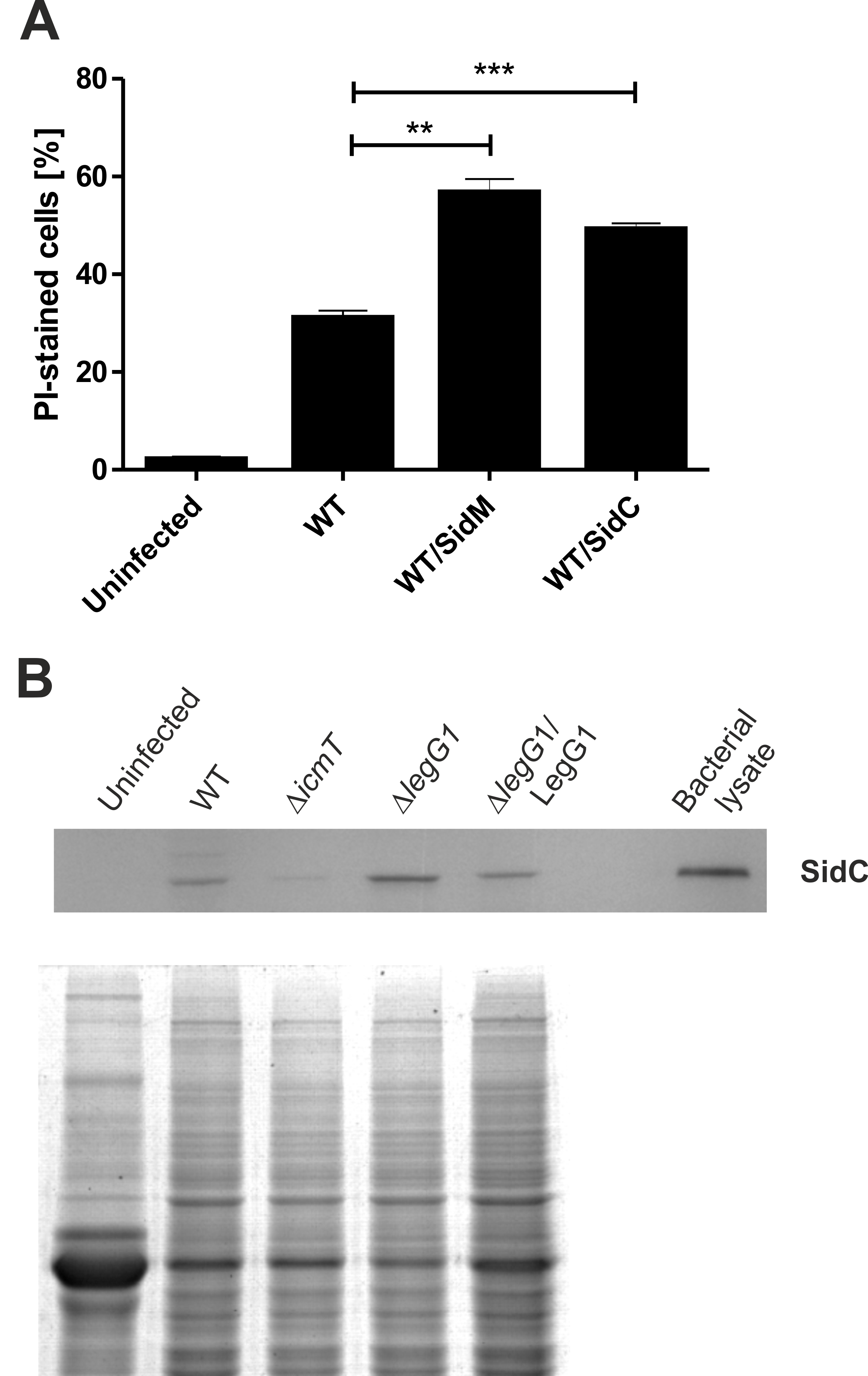

Supplement: Figure S6 — Toxicity and effector translocation of L. pneumophila ΔlegG1 strains overproducing SidM or SidC. (A) For toxicity assays RAW264.7 macrophages were infected (MOI 10, 4 h) with L. pneumophila wild-type harboring pCR033 (vector), pCR034 (M45-SidC) or pEB201 (M45-SidM), detached from the wells by scraping, stained with propidium iodide (1 µg/ml) and analyzed by flow cytometry. (B) To assay translocation efficiency, HeLa cells were infected (MOI 100, 1 h) with L. pneumophila wild-type, ΔicmT or ΔlegG1 harboring the vector pCR033, or with ΔlegG1/pSU19 (M45-LegG1), washed several times and lysed with 1% digitonin. 25 µl lysate of uninfected or infected HeLa cells or wild-type L. pneumophila were separated by SDS PAGE and stained with Coomassie Brilliant Blue, or were subjected to Western blot using an anti SidC antibody to quantify the amount of translocated effector protein. (TIF) [file ppat.1003598.s006.tif]

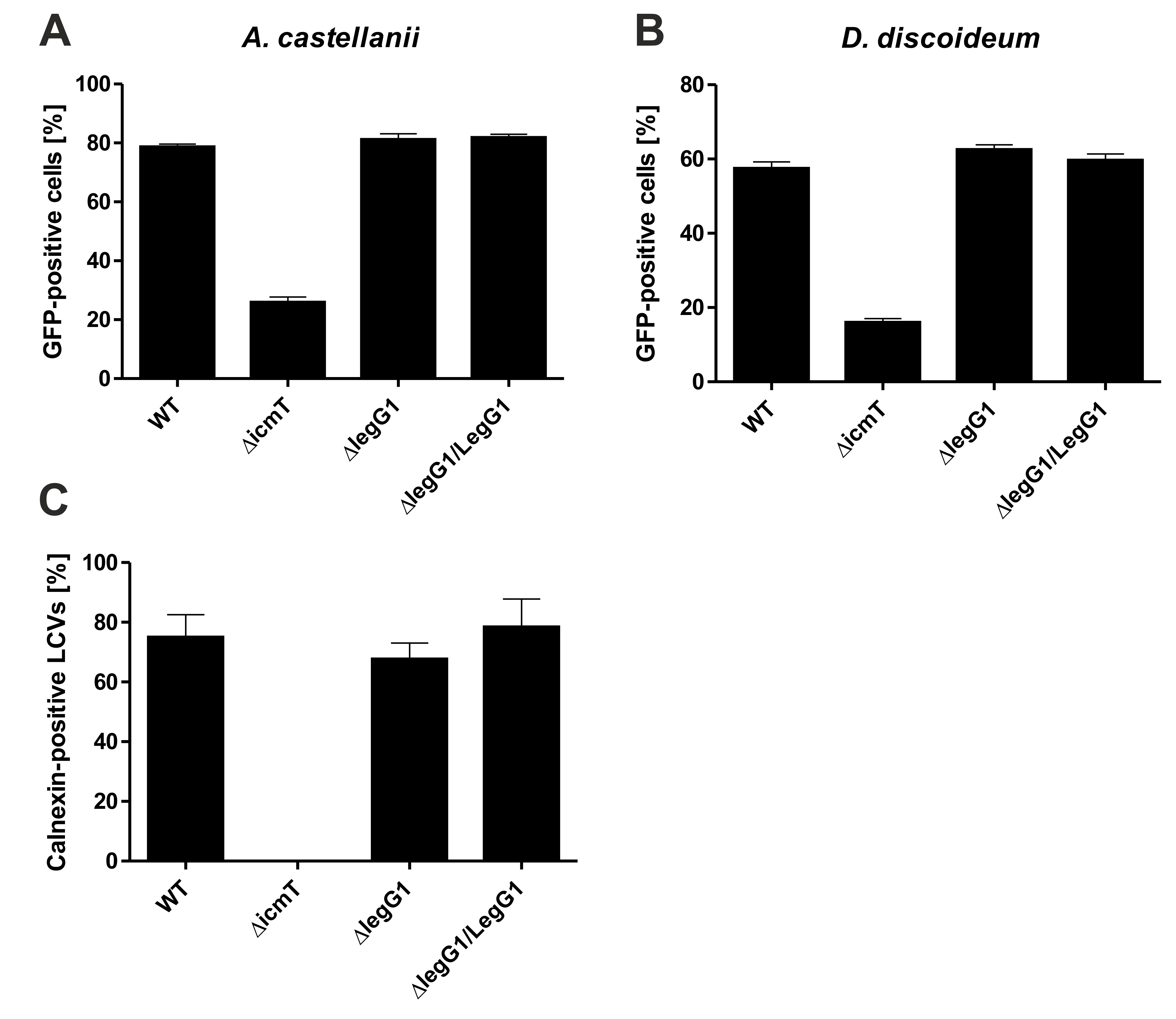

Supplement: Figure S7 — Uptake and LCV formation of L. pneumophila ΔlegG1. L. pneumophila ΔlegG1 is not impaired for uptake and LCV formation. (A) A. castellanii or (B) D. discoideum was infected (MOI 20, 45 min) with GFP-producing L. pneumophila wild-type, ΔlegG1 or ΔicmT harboring pCR076, or with ΔlegG1/pER4 (M45-LegG1), and uptake was determined by flow cytometry. (C) D. discoideum producing calnexin-GFP was infected (MOI 50, 1 h) with DsRed-producing L. pneumophila wild-type, ΔlegG1 or ΔicmT harboring pCR077, or with ΔlegG1/pER5 (M45-LegG1). The percentage of calnexin-GFP-positive LCVs (n = 100/strain, 4 independent experiments) was scored in lysates of infected cells. (TIF) [file ppat.1003598.s007.tif]

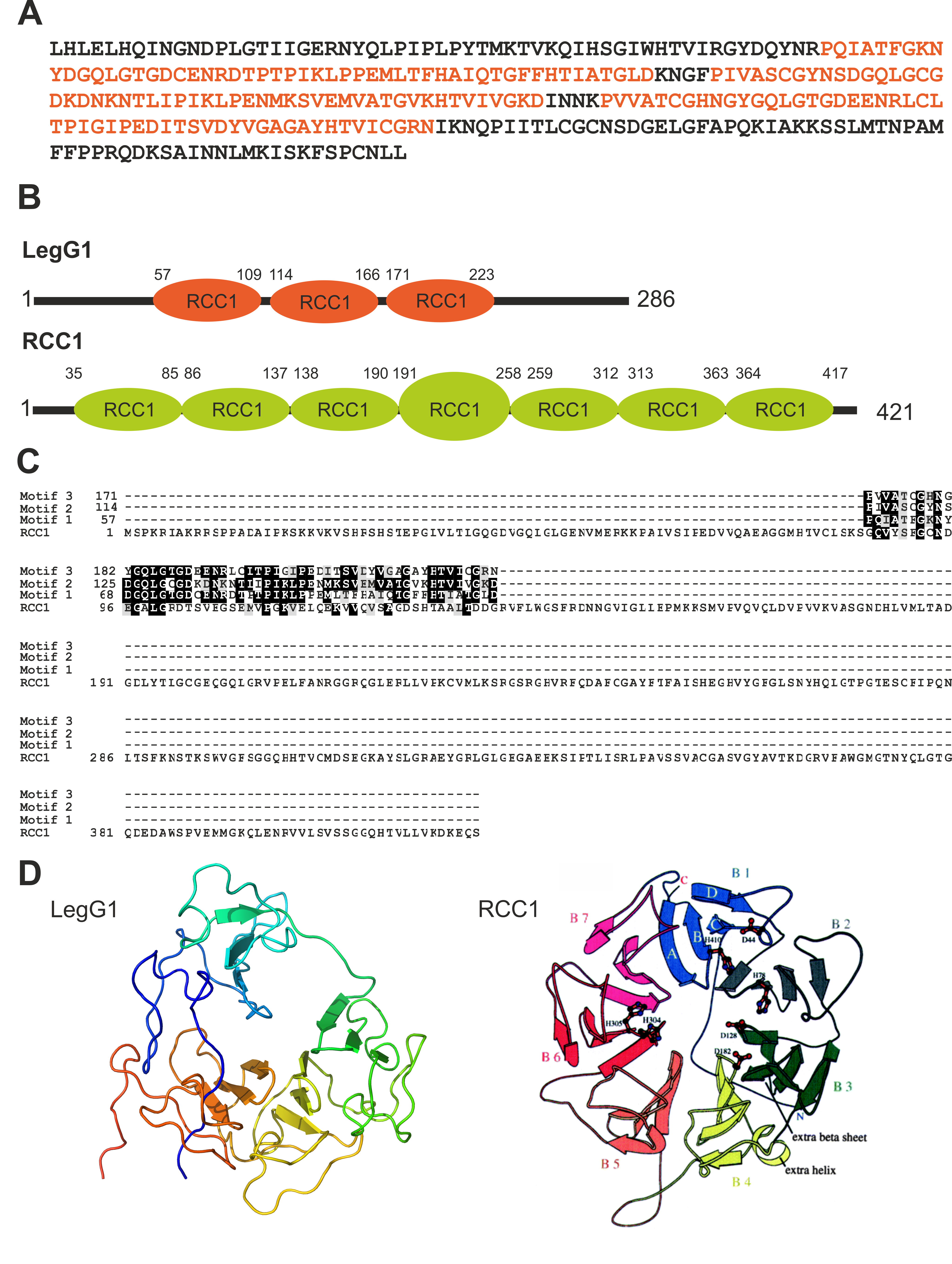

Supplement: Figure S8 — Comparison of L. pneumophila LegG1 with human RCC1. (A) Amino acid sequence of the 31.2 kDa L. pneumophila protein LegG1/Lpg1976 (286 amino acids). The three RCC1 domains, which are predicted by the PROSITE program (http://prosite.expasy.org/), are highlighted in red. (B) Schematic overview and position of RCC1 domains in L. pneumophila LegG1 and human RCC1 Ran GEF. (C) Alignment of the three RCC1 domains of LegG1 with a single RCC1 domain of RCC1. (D) Predicted structure of LegG1 (Phyre2; http://www.sbg.bio.ic.ac.uk/phyre2) and comparison with the X-ray crystallography structure at 1.7 Å resolution of human RCC1 forming a seven-bladed propeller (Renault et al. (1998) Nature 392: 97–101). (TIF) [file ppat.1003598.s008.tif]
