# Supplementary material for: Activation of Ran GTPase by a Legionella Effector Promotes Microtubule Polymerization, Pathogen Vacuole Motility and Infection
Source: PLoS Pathog. 2013 Sep 19;9(9):e1003598. doi: 10.1371/journal.ppat.1003598 (PMC3777869; doi:10.1371/journal.ppat.1003598)
Supplement: Table S1 — Strains and plasmids. (DOCX) [file ppat.1003598.s011.docx]

**Supplementary Table S1.** Strains and plasmids.

| **Strain/plasmid** | **Relevant properties ^a^** | **Reference** |
| --- | --- | --- |
| *E. coli* |  |  |
| TOP10 |  | Invitrogen |
| BL21 (DE3) |  | Novagen |
| *L. pneumophila* |  |  |
| ER01 | JR32 *legG1*::Kan^R^ (Δ*legG1*) | This study |
| GS3011 | *L. pneumophila* JR32 *icmT3011*::Kan^R^ (Δ*icmT*) | [[1](#_ENREF_1)] |
| JR32 | Virulent *L. pneumophila* serogroup 1 strain Philadelphia | [[2](#_ENREF_2)] |
| *Y. enterocolitica* |  |  |
| WA (pT3SS) | WA-C/ mini pYV virulence plasmid (pT3SS); Spe^R^ | [[3](#_ENREF_3)] |
| *D. discoideum* |  |  |
| Ax3/GFP-tubulin | pDEXRH-*gfp*-*tubA* | [[4](#_ENREF_4)] |
| Ax3/pCaln-GFP | P*_act15_*, calnexinA-RSSSKLK-GFP (S65T), G418^R^ | [[5](#_ENREF_5)] |
| Ax3/pSU17 | DdRanA-GFP | This work |
| Ax3/pSU26 | DdRanBP1-GFP | This work |
| Plasmids |  |  |
| pCJYE53-G3 | *Sal*I-*Hin*dIII *yopE53-gfp3* fragment in pACYC184, Cam^R^ | [[6](#_ENREF_6)] |
| pCJYE138-G3 | *Sal*I-*Hin*dIII *yopE138-gfp3* fragment in pACYC184, Cam^R^ | [[6](#_ENREF_6)] |
| pCR2 | pGEX-4T-1-*sidC* | [[7](#_ENREF_7)] |
| pCR33 | *Legionella* expression vector, Δ*mobA*, RBS, M45-(Gly)_5_, Cam^R^, (= pMMB207-C-RBS-M45) | [[7](#_ENREF_7)] |
| pCR34 | pMMB207C-M45-*sidC* | [[7](#_ENREF_7)] |
| pCR76 | pMMB207C-P*_tac_*-RBS-*gfp*-RBS-MCS | [[8](#_ENREF_8)] |
| pCR77 | pMMB207C-P*_tac_*-RBS-*dsred*-RBS-MCS | [[8](#_ENREF_8)] |
| pDXA-HC | *Dictyostelium* expression vector, P*_act15_*, Neo^R^, Amp^R^ | [[9](#_ENREF_9)] |
| pEB189 | pGEX-4T-1-*sidM* | [[10](#_ENREF_10)] |
| pEB201 | pMMB207C-M45-*sidM* | [[10](#_ENREF_10)] |
| pER2 | pET-28a-*legG1*-His_6_ | This work |
| pER3 | pET-28a-His_6_-*legG1* | This work |
| pER4 | pCR76-M45-*legG1* | This work |
| pER5 | pCR77-M45-*legG1* | This work |
| pER35 | pET-28a-*legG1*(N223A)-His_6_ | This work |
| pET-28a(+) | Expression of N-terminal His-tag fusions; P_T7_; Kan^R^ | Novagen |
| pGEM-T easy | Cloning of PCR products, Amp^R^ | Promega |
| pGP3 | pCJYE53-G3-*legG1*, *Bam*HI*/Sal*I | This work |
| pGP4 | pCJYE138-G3-*legG1*, *Bam*HI*/Sal*I | This work |
| pGP7 | pCJYE53-G3-*sidC*, *Bam*HI*/Sal*I | This work |
| pGP8 | pCJYE138-G3-*sidC*, *Bam*HI*/Sal*I | This work |
| pGP9 | pCJYE53-G3-*sidM*, *Bam*HI*/Sal*I | This work |
| pGP10 | pCJYE138-G3-*sidM*, *Bam*HI*/Sal*I | This work |
| pGP11 | pCJYE53-G3-*sidC*_P4C, *Bam*HI*/Sal*I | This work |
| pGP12 | pCJYE138-G3-*sidC*_P4C, *Bam*HI*/Sal*I | This work |
| pGP29 | pCJYE53-G3-ΔGFP, *Bam*HI/*Sal*I + Klenow | This work |
| pGP30 | pCJYE138-G3-ΔGFP, *Bam*HI/*Sal*I + Klenow | This work |
| pHP56 | pGEX-4T-1-*sidC*_P4C | [[11](#_ENREF_11)] |
| pLAW344 | *oriT* (RK2), *oriR* (ColE1), *sacB*, Cam^R^, Amp^R^ | [[12](#_ENREF_12)] |
| pMMB207C | *Legionella* expression vector, Δ*mobA*, - RBS, Cam^R^ | [[7](#_ENREF_7)] |
| pNT28 | pMMB207C-RBS-*gfp* (constitutive *gfp*) | [[13](#_ENREF_13)] |
| pSU1 | pGEM-T-easy-up *legG1´*-Kan^R^-down ´*legG1* | This work |
| pSU2 | pLAW344-up *legG1´*-Kan^R^-down´*legG1* | This work |
| pSU17 | pDXA-HC-*ranA*-*gfp* | This work |
| pSU19 | pMMB207C-M45-*legG1* | This work |
| pSU26 | pDXA-HC-*ranBP1*-*gfp* | This work |
| pSW001 | pMMB207C, Δ*lacI*^q^ (constitutive *dsred*) | [[14](#_ENREF_14)] |
| pSW102 | MCS-*gfp* in pDXA | [[15](#_ENREF_15)] |
| pYopESycE | *yopE*-*sycE* PCR fragment in pACYC184 (*Hin*dIII) | [[3](#_ENREF_3)] |
| pXDC61-FabI | pMMB207C-*blaM*-*fabI* | [[16](#_ENREF_16)] |
| pXDC61-LegG1 | pMMB207C-*blaM*-*legG1* | [[16](#_ENREF_16)] |
| pXDC61-LepA | pMMB207C-*blaM*-*lepA* | [[16](#_ENREF_16)] |

^a^ Abbreviations: Amp, ampicillin; Cam, chloramphenicol; Kan, kanamycin; Gen, gentamicin; G418, geneticin, Spe, spectinomycin.

**Supplementary References**

1. Segal G, Shuman HA (1998) Intracellular multiplication and human macrophage killing by *Legionella pneumophila* are inhibited by conjugal components of IncQ plasmid RSF1010. Mol Microbiol 30: 197-208.

2. Sadosky AB, Wiater LA, Shuman HA (1993) Identification of *Legionella pneumophila* genes required for growth within and killing of human macrophages. Infect Immun 61: 5361-5373.

3. Trülzsch K, Roggenkamp A, Aepfelbacher M, Wilharm G, Ruckdeschel K, et al. (2003) Analysis of chaperone-dependent Yop secretion/translocation and effector function using a mini-virulence plasmid of *Yersinia enterocolitica*. Int J Med Microbiol 293: 167-177.

4. Neujahr R, Albrecht R, Kohler J, Matzner M, Schwartz JM, et al. (1998) Microtubule-mediated centrosome motility and the positioning of cleavage furrows in multinucleate myosin II-null cells. J Cell Sci 111: 1227-1240.

5. Müller-Taubenberger A, Lupas AN, Li H, Ecke M, Simmeth E, et al. (2001) Calreticulin and calnexin in the endoplasmic reticulum are important for phagocytosis. EMBO J 20: 6772-6782.

6. Jacobi CA, Roggenkamp A, Rakin A, Zumbihl R, Leitritz L, et al. (1998) *In vitro* and i*n vivo* expression studies of *yopE* from *Yersinia enterocolitica* using the *gfp* reporter gene. Mol Microbiol 30: 865-882.

7. Weber SS, Ragaz C, Reus K, Nyfeler Y, Hilbi H (2006) *Legionella pneumophila* exploits PI(4)*P* to anchor secreted effector proteins to the replicative vacuole. PLoS Pathog 2: e46.

8. Finsel I, Ragaz C, Hoffmann C, Harrison CF, Weber S, et al. (2013) The *Legionella* effector RidL inhibits retrograde trafficking to promote intracellular replication. Cell Host Microbe 14: 38-50.

9. Manstein DJ, Schuster HP, Morandini P, Hunt DM (1995) Cloning vectors for the production of proteins in *Dictyostelium discoideum*. Gene 162: 129-134.

10. Brombacher E, Urwyler S, Ragaz C, Weber SS, Kami K, et al. (2009) Rab1 guanine nucleotide exchange factor SidM is a major phosphatidylinositol 4-phosphate-binding effector protein of *Legionella pneumophila*. J Biol Chem 284: 4846-4856.

11. Ragaz C, Pietsch H, Urwyler S, Tiaden A, Weber SS, et al. (2008) The *Legionella pneumophila* phosphatidylinositol-4 phosphate-binding type IV substrate SidC recruits endoplasmic reticulum vesicles to a replication-permissive vacuole. Cell Microbiol 10: 2416-2433.

12. Wiater LA, Sadosky AB, Shuman HA (1994) Mutagenesis of *Legionella pneumophila* using Tn*903*dll*lacZ*: identification of a growth-phase-regulated pigmentation gene. Mol Microbiol 11: 641-653.

13. Tiaden A, Spirig T, Weber SS, Brüggemann H, Bosshard R, et al. (2007) The *Legionella pneumophila* response regulator LqsR promotes host cell interactions as an element of the virulence regulatory network controlled by RpoS and LetA. Cell Microbiol 9: 2903-2920.

14. Mampel J, Spirig T, Weber SS, Haagensen JAJ, Molin S, et al. (2006) Planktonic replication is essential for biofilm formation by *Legionella pneumophila* in a complex medium under static and dynamic flow conditions. Appl Environ Microbiol 72: 2885-2895.

15. Weber SS, Ragaz C, Hilbi H (2009) The inositol polyphosphate 5-phosphatase OCRL1 restricts intracellular growth of *Legionella*, localizes to the replicative vacuole and binds to the bacterial effector LpnE. Cell Microbiol 11: 442-460.

16. de Felipe KS, Glover RT, Charpentier X, Anderson OR, Reyes M, et al. (2008) *Legionella* eukaryotic-like type IV substrates interfere with organelle trafficking. PLoS Pathog 4: e1000117.
