# Supplementary material for: Activation of Ran GTPase by a Legionella Effector Promotes Microtubule Polymerization, Pathogen Vacuole Motility and Infection
Source: PLoS Pathog. 2013 Sep 19;9(9):e1003598. doi: 10.1371/journal.ppat.1003598 (PMC3777869; doi:10.1371/journal.ppat.1003598)
Supplement: Table S2 — Oligonucleotides used in this study. (DOCX) [file ppat.1003598.s012.docx]

**Supplementary Table S2.** Oligonucleotides used in this study.

| **Oligo** | **Sequence (5' - 3') ^a^** | **Comments** |
| --- | --- | --- |
| oCR149 | GCTGTTGACAATTAATCATCGG | pMMB207 for. (seq. primer) |
| oCR150 | CGTTCTGATTTAATCTGTATCAGGC | pMMB207 rev. (seq.primer) |
| oCR158 | AAAAACGCGGATCCTTGCATCTTGAATTGCATC | 5’ of *legG1*, *Bam*HI |
| oCR160 | AAAAACGCTCTAGACTATTATCATAACAAATTGCATGGCG | 3’ of *legG1*, *Xba*I |
| oER01 | CGGCAAACCTGATGCAACCC | 5’ of upstream region of *legG1* (seq. primer) |
| oER02 | GCCATGTTGCTCCGGTAACC | 3’ of downstream region of *legG* (seq. primer) |
| oER03 | AGACCAATGTCGACTCATAACAAATTGCATGGCG | 3’ of *legG1*, *Sal*I (pCJYE) |
| oER04 | AAAAAGTCGACTTATTAGTGATGATGATGATGATGGCCGCTGCCTAACAAATTGCATGGCGAGAATTTAC | 3´ of *legG1, Sal*I His_6_-Tag |
| oER05 | AAAAAGTCGACTTATTATAACAAATTGCATGGCGAGAATTTAC | 3` of *legG1*, *Sal*I, 2x Stop |
| oER06 | AAAAACCATGGAACATCTTGAATTGCATCAAATAAATGGCAATGACCC | 5` of *legG1*, *Nco*I |
| oER07 | AAAAACCCGGGATGGCCATGGATCGGAGTAGG | Insertion of M45-*legG1* into pCR076 and pCR077, *Sma*I |
| pER22 | GGCGCCTATCATACAGTCATTTGTGGGCGGGCTATAAAAAATCAGCCAATTATAACC | 5` quick change mutagenesis of *legG1* (N223A) |
| pER23 | GGTTATAATTGGCTGATTTTTTATAGCCCGCCCACAAATGACTGTATGATAGGCGCC | 3` quick change mutagenesis of *legG1* (N223A) |
| oER25 | AAAAAATGCATTTGCATCTTGAATTGCATCAAATAAATGG | 5' of *legG1*, *Nsi*I (pDXA-HC-GFP) |
| oER26 | AAAAAATGCATTCATAACAAATTGCATGGCGAGAATTTAC | 3' of *legG1*, *Nsi*I (pDXA-HC-GFP) |
| oER27 | AAAAAATGCATTCATGGCGAGAATTTACTAATTTTCATC | 3' of *legG1_*ΔCAAX, *Nsi*I (pDXA-HC-GFP) |
| oSU92 | AAAAGGTACCATGTCAGAAGTCGAAAAGAAAG | 5' of *RanBP1*-*gfp*, *Kpn*I |
| oSU93 | AAAAGGATCCTTCAGTTTTTTTTTCAATTGCTTC | 3' of *RanBP1*-*gfp*, *Bam*HI |
| oSU94 | AAGTTAGTTATCCTATCTAAGCTAAC | 5’ of upstream region of *legG1* (deletion) |
| oSU95 | AAAAGGATCCTTATTTGATGCAATTCAAGATGC | 3’ of upstream region of *legG1* (deletion), *Bam*HI |
| oSU96 | AAAAGGATCCGGTTTAATTGGTCTCCATAGAG | 5’ of downstream region of *legG1* (deletion), *Bam*HI |
| oSU97 | AAGGCAACCAGAAACCGGGC | 3’ of downstream region of *legG1* (deletion) |
| oSU104 | AAAAGGTACCATGGCAGAAAAAGAACAAATTAAATTAG | 5’ of *RanA*-*gfp*, *Kpn*I |
| oSU105 | AAAAGGATCCCAAGTCATCATTGTCTTCTGGTA | 3’ of *RanA*-*gfp*, *Bam*HI |
| oKan3’ | GAGTTTTCTCCTTCATTACAG | 5’ out of Kan^R^, deletion test |
| oKan5’ | CGATAGATTGTCGCACCTG | 3’ out of Kan^R^, deletion test |

^a^ Restriction sites are underlined.
