# Supplementary material for: Activation of Ran GTPase by a Legionella Effector Promotes Microtubule Polymerization, Pathogen Vacuole Motility and Infection
Source: PLoS Pathog. 2013 Sep 19;9(9):e1003598. doi: 10.1371/journal.ppat.1003598 (PMC3777869; doi:10.1371/journal.ppat.1003598)
Supplement: Table S3 — Oligonucleotides used for RNA interference. (DOCX) [file ppat.1003598.s013.docx]

**Supplementary Table S3.** Oligonucleotides used for RNA interference.

| **NCBI gene** | **Gene description** | **Entrez Gene ID** | **Product name** | **Product ID** |
| --- | --- | --- | --- | --- |
| ARF1 | ADP-ribosylation factor 1 | 375 | Hs_ARF1_1 (1) | SI00299250 |
|  |  |  | Hs_ARF1_10 (2) | SI02757272 |
|  |  |  | Hs_ARF1_11 (3) | SI02757279 |
|  |  |  | Hs_ARF1_8 (4) | SI02654470 |
| Ran | RAN, member RAS oncogene family | 5901 | Hs_RAN_10 (1) | SI04950519 |
|  |  |  | Hs_RAN_7 (2) | SI04950498 |
|  |  |  | Hs_RAN_8 (3) | SI04950505 |
|  |  |  | Hs_RAN_9 (4) | SI04950512 |
| RanBP1 | RAN binding protein 1 | 5902 | Hs_RANBP1_3 (1) | SI00698201 |
|  |  |  | Hs_RANBP1_4 (2) | SI00698208 |
|  |  |  | Hs_RANBP1_6 (3) | SI03188381 |
|  |  |  | Hs_RANBP1_7 (4) | SI04142089 |
